# *In silico* identification of papaya genome-encoded microRNAs to target begomovirus genes in papaya leaf curl disease

**DOI:** 10.3389/fmicb.2024.1340275

**Published:** 2024-03-21

**Authors:** Aarshi Srivastava, Vineeta Pandey, Nupur Singh, Avinash Marwal, Muhammad Shafiq Shahid, R. K. Gaur

**Affiliations:** ^1^Department of Biotechnology, Deen Dayal Updhyaya Gorakhpur University, Gorakhpur, India; ^2^Institute of Agriculture and Natural Sciences, Department of Biotechnology, Deen Dayal Updhyaya Gorakhpur University, Gorakhpur, India; ^3^Department of Biotechnology, Mohanlal Sukhadia University, Udaipur, India; ^4^Department of Plant Sciences, College of Agricultural and Marine Sciences, Sultan Qaboos University, Muscat, Oman

**Keywords:** begomovirus, *Carica papaya*, papaya leaf curl disease, secondary structure, microRNA

## Abstract

Papaya leaf curl disease (PaLCuD) is widespread and classified in the genus *begomovirus* (*Geminiviridae*), disseminated by the vector whitefly *Bemisia tabaci*. RNA interference (RNAi)-based antiviral innate immunity stands as a pivotal defense mechanism and biological process in limiting viral genomes to manage plant diseases. The current study aims to identify and analyze *Carica Papaya* locus-derived capa-microRNAs with predicted potential for targeting divergent begomovirus species-encoded mRNAs using a ‘four integrative *in silico* algorithms’ approach. This research aims to experimentally activate the RNAi catalytic pathway using *in silico*-predicted endogenous capa-miRNAs and create papaya varieties capable of assessing potential resistance against begomovirus species and monitoring antiviral capabilities. This study identified 48 predicted papaya locus-derived candidates from 23 miRNA families, which were further investigated for targeting begomovirus genes. Premised all the four algorithms combined, capa-miR5021 was the most anticipated miRNA followed by capa-miR482, capa-miR5658, capa-miR530b, capa-miR3441.2, and capa-miR414 ‘effective’ papaya locus-derived candidate capa-miRNA and respected putative binding sites for targets at the consensus nucleotide position. It was predicted to bind and target mostly to AC1 gene of the complementary strand and the AV1 gene of the virion strand of different begomovirus isolates, which were associated with replication-associated protein and encapsidation, respectively, during PaLCuD. These miRNAs were also found targeting betaC1 gene of betasatellite which were associated with retardation in leaf growth and developmental abnormalities with severe symptoms during begomovirus infection. To validate target prediction accuracy, we created an integrated Circos plot for comprehensive visualization of host–virus interaction. *In silico*-predicted papaya genome-wide miRNA-mediated begomovirus target gene regulatory network corroborated interactions that permit *in vivo* analysis, which could provide biological material and valuable evidence, leading to the development of begomovirus-resistant papaya plants. The integrative nature of our research positions it at the forefront of efforts to ensure the sustainable cultivation of papaya, particularly in the face of evolving pathogenic threats. As we move forward, the knowledge gained from this study provides a solid foundation for continued exploration and innovation in the field of papaya virology, and to the best of our knowledge, this study represents a groundbreaking endeavor, undertaken for the first time in the context of PaLCuD research.

## Introduction

Begomoviruses are a group of plant viruses that belong to the family *Geminiviridae*. Geminiviruses are circular, one-stranded non-enveloped DNA viruses with one (DNA-A, monopartite) or two (DNA-A and B, bipartite) 2.5–3.0 kb strands of DNA. This pathogen is widespread globally and poses a significant threat to numerous agricultural crops (Fiallo-Olivé et al., 2021). Begomoviruses have a circular, single-stranded DNA genome, and they are transmitted by whiteflies. It is known that begomoviruses rely heavily on their host plants for many aspects of their life cycle, including replication, transcription, and movement within the plant (Pandey et al., 2023). Begomoviruses have evolved intricate mechanisms to manipulate host cellular processes and evade plant defense responses, allowing them to establish successful infections (Nabity et al., 2009).

The genome of begomoviruses encodes for proteins (Supplementary Figure S1) that are involved in viral encapsidation (AV1/V1), movement (AV2/V2), and transcriptional activator protein (AC2/C2) (Saxena et al., 1998). In addition, DNA-A also encodes the replication-associated protein (Rep; AC1/C1), which plays a vital role, in viral DNA replication and interacts with host factors to ensure successful viral replication (Zaidi et al., 2017). The other accessory genes such as AC3 and C4 also play important roles in virus–host interactions and symptom development (Yazdani-Khameneh et al., 2016). The begomovirus are also associated with some sub-viral particle such as betasatellite that encodes for betaC1 protein has ATPase and DNA binding functions that regulates the accumulation of helper virus and are characterized as pathogenicity determinant, suppressing RNA silencing, while alphasatellite encodes for α-Rep protein found modulating plant development and physiology (Sivalingam et al., 2011).

The whitefly species complex is the carrier for multiple distinct species of the genus Begomovirus (Geminiviridae), including papaya leaf curl disease (PaLCuD). This disease can have significant negative effects on papaya plant health and crop productivity (Srivastava et al., 2022a). The disease is characterized by the curling and distortion of the leaves, stunted growth, and reduced fruit yield. PaLCuD infection has resulted in significant yield losses, which range from 40 to 100% which has been reported in the United States and Mexico (Alabi et al., 2016; Alcala-Briseno et al., 2020; Srivastava et al., 2023). Infected plants may also exhibit yellowing of the leaves and a general decline in health. The severity of symptoms can vary depending on the virus strain, the host plant species, and environmental conditions (Pandey et al., 2021). Managing PaLCuD is challenging due to the genomic complexities of the begomoviruses, the diversity of viral strains, and the wide range of hosts and vectors (Pandey et al., 2022; Srivastava et al., 2022b). Current management strategies involve several implementations related to integrated pest management strategies. These involve whitefly control, planting resistant varieties, removing, and destroying infected plants, proper cultural practices, monitoring, and early detection (Srivastava et al., 2023).

The regulation of various classes of microRNAs (miRNAs) has become crucial in governing plant growth and development, emerging as significant gene regulators (Adjei et al., 2021). A fresh realization entails double-stranded RNA-mediated interference (RNAi) is a biological response to double-stranded RNA that has evolved to be a conserved evolutionary strategy. It controls the expression of genes that code for proteins, mediates resistance to endogenous parasitic and exogenous pathogenic nucleic acids, and is a highly successful innate defense mechanisms against plant viral replication and/or translation (Ashraf et al., 2023). Among various classes of small endogenous RNA 92 molecules such as small transfer RNA (tRNA), ribosomal RNA (rRNA), small nucleolar RNA (snoRNA), small interfering RNA (siRNA), and microRNA (miRNA), miRNAs have been identified as notably more diverse and functionally active through biochemical and functional analyses (Reinhart et al., 2002).

MicroRNAs, also known as small non-coding endogenous RNA regulatory molecules, are a class of molecules with 20–24 nucleotides (nt) that are important for controlling gene expression in a variety of organisms, including plants, animals, and even certain viruses. Nuclear-encoded MIR genes produce the lengthy single-standard primary miRNAs (pri-miRNAs), which control gene regulation, cell growth, and host–virus interactions (Trobaugh and Klimstra, 2017) which means they influence the translation of messenger RNA (mRNA) into proteins or affect mRNA stability through post-transcriptional gene regulation (Liu et al., 2017a).

The generation of pre-miRNA, dispensation into stem-loop structures, advancement of intermediate duplexes (miRNA/miRNA*), stabilization of miRNA by 2-o-methylation, integration into RNA-induced silencing complexes (RISCs), and miRNA deprivation are all steps in the multifaceted multistep course known as miRNA biogenesis. This process also involves transcription, processing, and nuclear export. RISC breaks down the complementary target mRNA (Liu et al., 2017b). Hence, an innate defense mechanism known as microRNA-mediated RNA interference (RNAi) is capable of acting as a major defense strategy in retort to host–virus interactions. As a result, RNAi is an intrinsic mechanism that prevents virus infection at the post-transcriptional level (Mengistu and Tenkegna, 2021; Deng et al., 2022).

Using four *in silico* computations, the aim of the present investigation was to detect and analyze papaya genome-encoded microRNAs (capa-miRNA) that are expected to target begomovirus-encoded mRNAs: C-mii, *psRNATarget*, Tapirhybrid, and RNA22. The RNA interference (RNAi) system involves proteins that cleave or suppress an intended mRNA transcript when miRNA interacts with it in a sequence-specific manner (Agrawal et al., 2003; Wang et al., 2012).

The intention is to use the anticipated miRNAs to initiate RNA interference and produce papaya trees that are resistant to Begomovirus. These miRNAs have a major impact on the control of development processes, biotic and abiotic stress feedback, and the growth and development of plant cells as well as their ability to differentiate (Guleria and Yadav, 2011; D’Ario et al., 2017). To assess host–virus relationships in light of environmental stressful conditions, such as virus infection, regulatory network assessment of the plant in question is a crucial first step. This includes the recognition, evaluation, and *in silico* validation of mature miRNAs in papaya (Chen et al., 2015; Yawichai et al., 2019).

In this study, a consolidative *in silico* approach for combatting divergent begomovirus genes was applied to predict papaya locus-derived capa-miRNAs that could target genes of begomovirus associated with PaLCuD. It could be used as a starting point to comprehend how host miRNAs regulate viral gene expression and open the door to developing defense mechanisms for begomovirus disease in papaya (*C. papaya*) plants. To the greatest extent of current understanding, this is a landmark study to identify putative capa-miRNAs that could target key genes in the genetic makeup of the begomovirus and its satellites.

## Materials and methods

### *Carica papaya* biological data retrieval

Sequence Read Archive (SRA) data provide raw sequencing data and alignment information generated with the help of high-throughput sequencing data from various metagenomic and environmental surveys. The RNA-Seq reads, i.e., BioSample: SAMN06673892; Sample name: Papaya_leaf_RNA-seq_1; SRA: SRS2092156 of *Carica papaya,* were downloaded from the National Center for Biotechnology Information (NCBI) database.^1^ The following RNA-Seq reads were filtered for leaf tissue and the maximum miRNA provided. According to the NCBI database, the experimental setting of the mRNA sequencing was carried out using RNA-Seq of *Carica papaya*: leaf with 1 ILLUMINA (HiSeq X Ten) run.

### Processing of assembly and quality check

The downloaded SRA data were analyzed, filtered, and assembled by the rnaviralSPAdes [*de novo* assembler for transcriptomes, metatranscriptomes, and metaviromes (ref) through Galaxy Version 3.15.4 (Galaxy^2^)]. QUAST (Gurevich et al., 2013) carried out the statistical analysis of the produced assembly (Table 1), which was then utilized to look for miRNA genes.

**Table 1:** Statistical analysis and quality check of Papaya_leaf_RNA-seq_1; SRA: SRS2092156 using QUAST.

naviralSPAdes_on_data_53__Contigs
# contigs (≥ 0 bp) 53318
# contigs (≥ 1,000 bp) 10615
Total length (≥ 0 bp) 35446602
Total length (≥ 1,000 bp) 19679411
# contigs 19118
Largest contig 12834
Total length 25731924
GC (%) 42.70
N50 1613
N90 699
auN 1901.0
L50 5276
L90 14750
# N’s per 100 kbp 0.00

### Begomovirus sequence data as target (isolates)

For this investigation, the whole genomes of newly identified 11 begomovirus isolate that cause papaya leaf curl diseases were used. The FASTA format was retrieved from the NCBI GenBank database^3^ of the National Center for Biotechnology Information. Begomoviruses linked to PaLCuD were newly identified in this study, sequenced, and uploaded to the GenBank database of the National Center for Biotechnology Information with the corresponding accession number (Table 2).

**Table 2:** Features of present sequenced papaya crop begomovirus isolates.

Sample	Begomoviruses and associated component				Accession no.	Isolate
	DNA-A	*DNA-B*	*Betasatellite*	*Alphasatellite*		
PL 1	*Papaya Leaf Curl Virus (PaLCuV)*	*_*	*Papaya Leaf Curl Betasatellite (PaLCuB)*	*_*	MZ605904 - MZ605905	Gorakhpur_av1
PL 6	*Papaya Leaf Curl Virus (PaLCuV)*	*_*	*Papaya Leaf Curl Betasatellite (PaLCuB)*	*_*	MZ669217 - MZ606364	Gorakhpur_av2
PL 10	*Cotton Leaf Curl Multan Virus(CLCuMuV)*	*_*	*Tomato Leaf Curl Bangladesh Betasatellite (ToLCBDB)*	*Papaya Leaf Curl Vishakapuri Alphasatellite (PaLCVSA)*	OQ440383 - OQ440384 - OQ440385	Bastar_RAV
PL 13	*Papaya Leaf Curl Virus (PaLCuV)*	*_*	*Tomato Leaf Curl Bangladesh Betasatellite (ToLCBDB)*	*_*	OR489166 - OQ440377	Delhi_RAV
PL 20	*Papaya Leaf Curl Virus (PaLCuV)*	*Tomato Leaf Curl New Delhi Virus(ToLCNDV)*	*_*	*_*	OQ134774 - OQ134775	Durg_RAV
PL 27	*Croton Yellow Vein Mosaic Virus (CYVMV)*	*_*	*Papaya Leaf Curl Betasatellite (PaLCuB)*	*_*	OQ168370 - OQ168371	Kahlilabad_RAV
PL 29	*Cotton Leaf Curl Virus (CLCuV)*	*_*	*Cotton Leaf Curl Betasatellite (CLCuB)*	*_*	OQ091756 - OQ091757	Maharajganj_RAV
PL 31	*Tomato Leaf Curl New Delhi Virus(ToLCNDV)*	*_*	*Tomato Leaf Curl Betasatellite (ToLCB)*	*_*	OQ290944 - OQ290945	Nautanwa_RAV
PL 36	*Papaya Leaf Curl Virus (PaLCuV)*	*_*	*Papaya Leaf Curl Betasatellite (PaLCuB)*	*_*	OQ290942 - OQ290943	Raipur_RAV
PL 43	*Papaya Leaf Curl Virus (PaLCuV)*	*_*	*Papaya Leaf Curl Betasatellite (PaLCuB)*	*Papaya Leaf Curl Alphasatellite (PaLCuA)*	OQ440378 - OQ440379 -OQ440380	Bilaspur_RAV
PL 45	*Tomato Leaf Curl New Delhi Virus(ToLCNDV)*	*_*	*Cotton Leaf Curl Betasatellite (CLCuB)*	*_*	OQ440381 - OQ440382	Mahasamund_RAV

### Prediction of potential miRNA and their secondary structures

Finding gene regulatory networks regulated by miRNA starts with the *in silico* prediction of miRNA-mRNA target sites. The C-mii tool (version 1.11) was used to identify targets and miRNA (Numnark et al., 2012). Using BLASTN (e-value cutoff: 10), the putative miRNA candidates were validated by scanning them against the published miRNAs of all the reference plants in miRBase. Using the UniProtKB/Swiss-Prot (plant only) (release 2010_12) and UniProtKB/TrEMBL (version 2011_01) protein databases, BLASTX (e-value <1e−5) OSC bioinformatic resources [The statistics of sequence analogy score, NCBI] were used to exclude the protein-coding regions. Other non-coding RNA databases were eliminated using RNA database Rfam 10.^4^ Rfam is a collection of many covariance models and sequence alignments that characterize RNA families without protein coding. These websites allow users to search a library of covariance models for a query sequence and examine a variety of sequence alignments and family annotations (Griffiths-Jones et al., 2006). The folding of primary and precursor miRNAs was done using UNAFold. The maximum base pair distance of 3,000, the maximum bulge/internal loop size of 30, and the single thread run at 37°C were the UNAFold settings that were used.

The miRNA identification tool employs a homology screening methodology. Sub-modules were employed to define default parameters for both primary and precursor miRNA folding. The stability of the pre-miRNAs’ secondary structure must be assessed to interpret the anticipated results. If a contig meets any of the following requirements, it is regarded as a miRNA candidate: (1) The length of the predicted miRNAs should be between 19 and 25 nucleotides; (2) the predicted mature miRNAs were permitted a maximum of five to six mismatches toward the reference miRNA; (3) the mature miRNA was localized inside the stem-loop structure with one arm; (4) the percentage of G + C and A + U content; and (5) the secondary structure’s minimal folding free energy (MFE) and high MFE index (MFEI) value ought to be highly negative (Singh et al., 2016a,b).

### Potential miRNA-target prediction in genomic regions of present begomovirus isolates

There are numerous computational approaches that can be used to identify viral mRNA’s putative miRNA-target locations that are advantageous for silencing. Specific criteria for miRNA prediction were established by each computational algorithmic tool. The accuracy of miRNA-target site prediction can be influenced by several factors, such as the specificity and sensitivity of the algorithm, the choice of reference sequence, and the length of the target sequence. A computational approach refers to the use of multiple computational methods, algorithms, or tools to analyze and interpret biological data. From the pathogenic begomoviruses isolates, the “most efficacious” miRNA-target sites of the papaya miRNAs have been identified and predicted using the C-mii tool, RNA22, Tapirhybrid, and psRNATarget programs. The predefined conditions were utilized for the analysis of the predicted transcripts (in FASTA format) and the capa-miRNA sequences generated from the papaya locus.

#### C-mii tool

Absolute or nearly ideal complementarities between plant miRNA and its target are the foundation of the C-mii target recognition module. Target scanning was done against the default settings to look for the complementary site of projected miRNAs for all filtered contigs (Singh and Sharma, 2014). C-mii established a number of standards for the prediction of miRNA target genes: (1) The target gene and projected mRNAs should not have more than four mismatches; (2) the complementary site’s tenth and eleventh positions should not have any mismatches; (3) the miRNA and target duplex’s MFE should be negative; and (4) the complimentary alignment should have no more than five GU pairs. Using RNAfold,^5^ the pre-miRNAs’ MFE was assessed.

#### psRNATarget

Employing complementary scoring criteria, the psRNATarget algorithm is a web-based, highly accurate plant miRNA prediction system (Dai and Zhao, 2011; Dai et al., 2018) that finds the target binding sites of plant miRNAs. The method forecasts the plant miRNAs’ inhibitory pattern of cleavage. The psRNATarget web server, accessible online at http://plantgrn.noble.org/psRNATarget, loaded the begomovirus genome’s FASTA sequence and predicted capa-miRNAs (accessed on February 25, 2023). The default criteria, which included an expected cutoff value of 7.0 and a method of inhibition set to “cleavage,” were used to predict the miRNA–mRNA target binding sites.

#### Tapirhybrid

The Tapirhybrid algorithm, which was created for seed and sequence-based predictions and recognizes miRNA-target relationships, is an online, swift, and accurate plant miRNA-target prediction algorithm (Bonnet et al., 2010). It can provide precise miRNA-target predictions, involving target mimics, and has FASTA and RNAhybrid search alternatives. The algorithm can be discovered via the Internet at http://bioinformatics.psb.ugent.be/webtools/tapir (accessed on March 12, 2023). Throughout this investigation, the normal baseline conditions (score < 9 and MFE ratio < 0.2) were applied.

#### RNA22

Accessible via the Internet at http://cm.jefferson.edu/rna22v1.0/, the RNA22 program is a versatile, web-based method that employs a pattern-recognition-based methodology (retrieved July 7, 2023) (Miranda et al., 2006). Using minimum folding energy (MFE), site complementarity, and non-seed-based interaction, it predicts statistically noteworthy target configurations (Loher and Rigoutsos, 2012). The predefined settings chosen for the purpose of identifying the miRNA-target binding sites of capa-miRNAs in the divergent isolates of the begomovirus sequence were output format (heteroduplexes); the highest possible folding energy for a heteroduplex was set at −15.00 Kcal/mol, and the levels of specificity and sensitivity were 63 and 61%, respectively.

#### Functional annotation of miRNA-target transcript

C-mii was used to functionally annotate the potential target transcripts using the UniProt/Swiss-Prot (plants only) database. Target transcripts that were shown to be successful in targeting the begomovirus gene were chosen for the purpose of functional annotation after evaluation. To identify the co-regulated targets by miRNA families and to rank the miRNA-targets according to MFE value, a biological connection was built among the discovered miRNA and the target it regulates. This networking was performed only for those miRNAs which were found to target begomovirus gene identified by all four-algorithm. Utilizing Cytoscape 3.2 software (Su et al., 2014), the biological network of miRNAs and their intended targets were displayed. Moreover, Circos plotting^6^ was performed to study a comprehensive visualization of host–virus interaction based on algorithm which was found targeting all the begomovirus genes.

#### Free energy (ΔG) estimation of duplex binding

RNAcofold is a new approach that uses the base-pairing patterns of miRNA-mRNA target duplex molecules and the minimal free energy to estimate the co-folding free energy (ΔG) of RNA duplex sequences. It is used especially to assess the duplex relationships among mRNA and miRNA. The RNAcofold web application (accessible publicly at http://rna.tbi.univie.ac.at/cgi-bin/RNAWebSuite/RNAcofold.cgi) has been configured with the FASTA sequences of the twin pair obtained from the psRNATarget study (accessed on September 19, 2023) (Bernhart et al., 2006).

## Results

### miRNA identification and characterization

**Figure 1:**
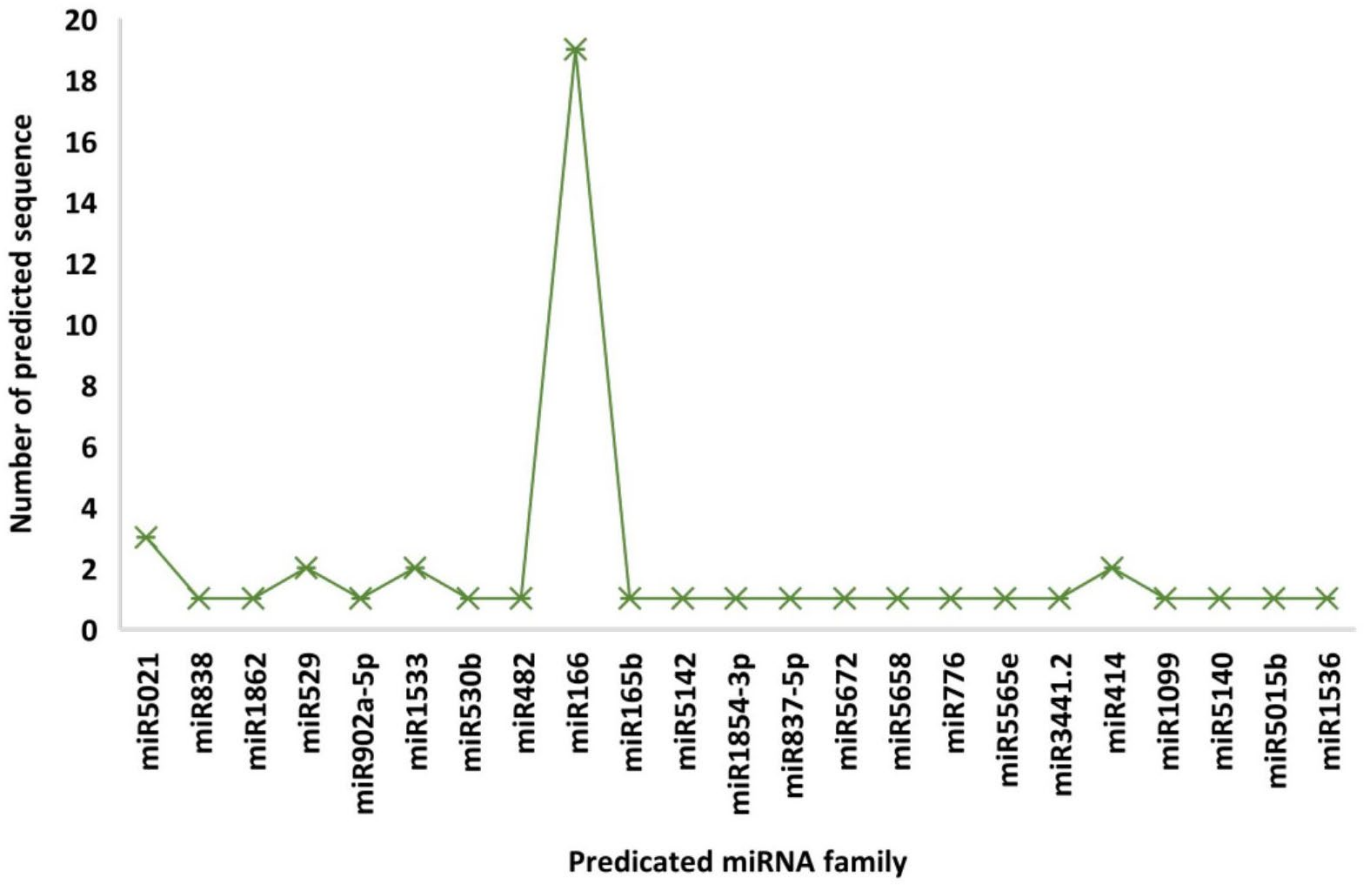
Number of predicted sequences for each microRNA family.

A total of 48 predicted candidates of 23 miRNA families (Figure 1) were obtained and filtered using different criteria (Table 3). Out of 48, 34 members were predicted to have the single transcript whereas rest 14 families were present in more than one transcript. Furthermore, very negative MFEI with transcripts was taken into consideration for the purpose of the research to lower false positive outcomes and increase accuracy. A wide variety of nucleotide lengths was seen in the majority of the projected mature miRNA sequences, and the distribution of A, U, G, and C content was not consistent. The secondary structures of only those predicted miRNAs are presented which have been found actively targeting begomovirus gene via all four algorithms.

**Table 3:** Salient parameters of predicted papaya locus-derived capa-miRNAs identified using C-mii tool.

Predicated miRNA family	Homolog miRNA	Predicted miRNA sequence	Sequence length	Nucleotide content	GC content (%)	AU content (%)	MFE (kcal/mol)	MFEI (kcal/mol)
miR5021	ath-miR5021	5′:AAAGAAGAAGAAGAAGAAGA:3′	549	A(136), U(169), G(122), C(122), N(0)	44.44	55.56	−140.9	−0.57
miR5021	ath-miR5021	5′:UGACGAGAAGAAGAAGAGAG:3′	93	A(30), U(27), G(19), C(17), N(0)	38.70	61.30	−28.5	−0.79
miR5021	ath-miR5021	5′:UAUGUAGAAGAAGAAGAAGA:3′	147	A(36), U(63), G(24), C(24), N(0)	32.65	67.35	−30.2	−0.62
miR838	aly-miR838	5′:UCUUCUUCUUCUUCUUCUUCA:3′	123	A(49), U(28), G(22), C(24), N(0)	37.39	62.61	−27.5	−0.59
miR1862f	osa-miR1862f	5′:CUUAGGUUGGUUUCUUUUAG:3′	95	A(29), U(33), G(19), C(14), N(0)	34.73	65.27	−26.2	−0.79
miR1862g	osa-miR1862g	5′:CUUAGGUUGGUUUCUUUUAG:3′	95	A(29), U(33), G(19), C(14), N(0)	34.73	65.27	−26.2	−0.79
miR529	bgy-miR529	5′:GAAGAAGAGAGAAGGAAGAA:3’	129	A(40), U(46), G(30), C(13), N(0)	33.33	66.67	−29.1	−0.67
miR529	bcy-miR529	5′:GAAGAAGAGAGAAGGAAGAA:3′	129	A(40), U(46), G(30), C(13), N(0)	33.33	66.67	−29.1	−0.67
miR902a-5p	ppt-miR902a-5p	5′:UGUCUUGGAGAUUCUUCA:3′	33	A(9), U(13), G(7), C(4), N(0)	33.33	66.67	−6.9	−0.62
miR1533	gma-miR1533	5′:AUGAUGAUAAUAAUAAUAA:3′	57	A(25), U(21), G(3), C(8), N(0)	19.29	80.71	−7.9	−0.71
miR1533	gma-miR1533	5′:AGUAGAAAGAUAAUAAUGA:3′	143	A(41), U(63), G(28), C(11), N(0)	27.27	72.73	−24.3	−0.62
miR530b	ptc-miR530b	5′:UGUUUUUGCAUCUGCAUCAU:3′	65	A(23), U(19), G(10), C(13), N(0)	35.38	64.62	−11.7	−0.50
miR482	zma-miR482	5’:UCUUCCUUGUUCUUCCAUGU:3’	1,432	A(424), U(355), G(311), C(342), N(0)	45.60	54.40	−333.7	−0.51
miR166i	zma-miR166i	5’:UCGGACCAGUCUUCUUCCAC:3′	49	A(16), U(14), G(9), C(10), N(0)	38.77	61.23	−10.2	−0.53
miR166h	zma-miR166h	5′:UCGGACCAGUCUUCUUCCAC:3′	49	A(16), U(14), G(9), C(10), N(0)	38.77	61.23	−10.2	−0.53
miR166g	zma-miR166g	5′:UCGGACCAGUCUUCUUCCAC:3′	49	A(16), U(14), G(9), C(10), N(0)	38.77	61.23	−10.2	−0.53
miR166f	zma-miR166f	5′:UCGGACCAGUCUUCUUCCAC:3′	49	A(16), U(14), G(9), C(10), N(0)	38.77	61.23	−10.2	−0.53
miR166e	zma-miR166e	5′:UCGGACCAGUCUUCUUCCAC:3′	49	A(16), U(14), G(9), C(10), N(0)	38.77	61.23	−10.2	−0.53
miR166d	zma-miR166d	5′:UCGGACCAGUCUUCUUCCAC:3′	49	A(16), U(14), G(9), C(10), N(0)	38.77	61.23	−10.2	−0.53
miR166c	zma-miR166c	5′:UCGGACCAGUCUUCUUCCAC:3′	49	A(16), U(14), G(9), C(10), N(0)	38.77	61.23	−10.2	−0.53
miR166b	zma-miR166b	5′:UCGGACCAGUCUUCUUCCAC:3′	49	A(16), U(14), G(9), C(10), N(0)	38.77	61.23	−10.2	−0.53
miR166b	vvi-miR166b	5′:UCGGACCAGUCUUCUUCCA:3′	49	A(16), U(14), G(9), C(10), N(0)	38.77	61.23	−10.2	−0.53
miR166a	vvi-miR166a	5′:UCGGACCAGUCUUCUUCCA:3′	49	A(16), U(14), G(9), C(10), N(0)	38.77	61.23	−10.2	−0.53
miR166b	tcc-miR166b	5′:UCGGACCAGUCUUCUUCCAC:3′	49	A(16), U(14), G(9), C(10), N(0)	38.77	61.23	−10.2	−0.53
miR166j	sbi-miR166j	5′:UCGGACCAGUCUUCUUCCAC:3′	49	A(16), U(14), G(9), C(10), N(0)	38.77	61.23	−10.2	−0.53
miR166i	sbi-miR166i	5′:UCGGACCAGUCUUCUUCCAC:3′	49	A(16), U(14), G(9), C(10), N(0)	38.77	61.23	−10.2	−0.53
miR166h	sbi-miR166h	5′:UCGGACCAGUCUUCUUCCAC:3′	49	A(16), U(14), G(9), C(10), N(0)	38.77	61.23	−10.2	−0.53
miR166d	sbi-miR166d	5′:UCGGACCAGUCUUCUUCCAC:3′	49	A(16), U(14), G(9), C(10), N(0)	38.77	61.23	−10.2	−0.53
miR166c	sbi-miR166c	5′:UCGGACCAGUCUUCUUCCAC:3′	49	A(16), U(14), G(9), C(10), N(0)	38.77	61.23	−10.2	−0.53
miR166b	sbi-miR166b	5′:UCGGACCAGUCUUCUUCCAC:3′	49	A(16), U(14), G(9), C(10), N(0)	38.77	61.23	−10.2	−0.53
miR166a	sbi-miR166a	5′:UCGGACCAGUCUUCUUCCAC:3′	49	A(16), U(14), G(9), C(10), N(0)	38.77	61.23	−10.2	−0.53
miR166c	csi-miR166c	5′:UCGGACCAGUCUUCUUCCAC:3′	49	A(16), U(14), G(9), C(10), N(0)	38.77	61.23	−10.2	−0.53
miR165b	aly-miR165b	5′:UCGGACCAGUCUUCUUCCAC:3′	49	A(16), U(14), G(9), C(10), N(0)	38.77	61.23	−10.2	−0.53
miR165a	aly-miR165a	5′:UCGGACCAGUCUUCUUCCAC:3′	49	A(16), U(14), G(9), C(10), N(0)	38.77	61.23	−10.2	−0.53
miR5142	rgl-miR5142	5′:UUGAUGAUAGAUAAGUGAU:3′	49	A(9), U(29), G(8), C(3), N(0)	22.44	77.56	−6.9	−0.62
miR1854-3p	osa-miR1854-3p	5’:UCGAUUUUGGGGAUUUGGUGAA:3′	78	A(15), U(28), G(21), C(14), N(0)	44.87	55.13	−25.1	−0.71
miR837-5p	aly-miR837-5p	5′:CUUUGUUUUUUUUUUUUUUCU:3′	161	A(41), U(69), G(29), C(22), N(0)	31.67	68.33	−28.1	−0.55
miR5672	gma-miR5672	5′:CAGGUAAUUGGAAGAAAUGGA:3′	50	A(15), U(16), G(11), C(8), N(0)	38.0	62.0	−10.9	−0.57
miR5658	ath-miR5658	5′:AGGAUGAUGAUGAUGAUGAUG:3′	157	A(45), U(53), G(44), C(15), N(0)	37.57	62.43	−42.2	−0.71
miR776	ath-miR776	5′:CCUAAGUCUUAUAUUGAUGUA:3′	71	A(22), U(27), G(10), C(12), N(0)	30.98	69.02	−12.3	−0.55
miR5565e	sbi-miR5565e	5′:UUGUUUGGUUGUUGACUGA:3′	114	A(43), U(33), G(16), C(22), N(0)	33.33	66.67	−23.2	−0.61
miR3441.2	aly-miR3441.2	5′:UACCACUUCAUAUUCUUUGAU:3′	88	A(30), U(26), G(15), C(17), N(0)	36.36	63.64	−23.1	−0.72
miR414	osa-miR414	5′:UUAUCAUCAUCAUCAUCAUCA:3′	246	A(84), U(70), G(51), C(41), N(0)	37.39	62.61	−67.7	−0.73
miR414	ath-miR414	5′:UUAUCAUCAUCAUCAUCAUCA:3′	246	A(84), U(70), G(51), C(41), N(0)	37.39	62.61	−67.7	−0.73
miR1099	smo-miR1099	5′:UUUAGCAAUGGUGAAUAUGUC:3′	317	A(102), U(79), G(79), C(57), N(0)	42.90	57.1	−81	−0.59
miR5140	rgl-miR5140	5′:UUUGGUGAAGAUUUGGUU:3′	436	A(121), U(112), G(100), C(103), N(0)	46.55	53.45	−104.1	−0.51
miR5015b	ath-miR5015b	5′:UCUUUUGUUGUUGUUGUUGUU:3′	109	A(23), U(37), G(30), C(19), N(0)	44.95	55.05	−32.6	−0.66
miR1536	gma-miR1536	5′:GAGGAGAGACAGAUGUGUUUG:3′	623	A(198), U(143), G(146), C(136), N(0)	45.26	54.74	−151.9	−0.53

MFE is minimum free energy; MFEI is defined as free energy index.

### GC content

The primary structure of stem-loop hairpins is formed and stabilized by the pairing of three hydrogen bonds between G and C. According to this reasoning, a sequence with a substantial GC content must be stable in its secondary structure of RNA. In this study, predicted miRNA families show variable GC contents in their pre-miRNA sequence, and their range of GC content varied from 19.29 to 46.55 (Table 3). Previous study stated that in plant *Helianthus* and *Nicotiana tabacum,* the predicated miRNA families such as miRNA160 and miRNA164 are in rich GC content and regarded as most conserved miRNA families (Barozai et al., 2012). In contrast, the AU content ranged from 53.45 to 80.71 and was shown to be high in comparison with the GC level (Table 3). According to reports from *Gossypium arboretum* L. and *Brassica rapa* L., uracil was found to be prominent in the first position of the majority of mature miRNA sequences (Table 3), indicating its significant significance in miRNA-mediated regulatory in plants (Luo et al., 2013).

### MFE and MFEI

MFE can be used to assess the endurance of the secondary structure of nucleic acids, such as DNA and RNA. According to Bonnet et al. (2004), precursor microRNAs are said to have lesser folding energy than other non-coding RNAs. The MFE values of the predicted precursor miRNAs in this study, which were discovered to be incorporated into C-mii and predicted by the UNAfold software, were revealed to be extremely negative, ranging from 6.9 to 333.7 (−kcal/mol). Furthermore, using MFE alone for describing miRNA is insufficient because precursor miRNAs vary in sequence. To differentiate miRNA from RNAs, the MFEI resolution for length of variation was also computed (Zhang et al., 2006a). In *Carica papaya*, the anticipated pre-miRNAs’ MFEI ranged from 0.50 to 0.73 (−kcal/mol) (Table 3). To ensure the integrity of the anticipated miRNAs’ secondary structure, RNAfold assessed MFEI. The median value was 0.59, although both results had a strong resemblance, indicating the thermodynamic stability of secondary structure. The resulting values were found to be less than those of tRNA (0.64), mRNAs (0.62–0.66), and rRNA (0.59) (Zhang et al., 2006a,b) suggesting that these newly identified capa-miRNAs are likely to be true miRNAs. Pre-miRNAs have a secondary arrangement resembling a hairpin. Asymmetric bulges are typically formed by unpaired groupings of nucleotide bases. The robustness of the structure is represented by the minimum size of these bulges, which is a crucial characteristic of plant miRNAs. Not a single family in the research displays a bulge. Reduced bulge count and bulge size increased the likelihood of true positive identification of characterized miRNAs.

### Target prediction

#### *Carica papaya* locus-derived capa-miRNAs targeting different begomovirus isolates

The *C. papaya* locus-derived capa-miRNAs’ *in silico* predictions have the ability to target viral ORFs encoded by various strains of the begomovirus genome. Depending on the C-mii, psRNATarget, RNA22, and Tapirhybrid algorithms, the projected cleavable targets for papaya locus-derived capa-miRNAs targeting multiple locus locations in the various begomovirus genera were the predicted begomovirus gene sequences as targeted by the capa-miRNAs.

#### Association of capa-miRNAs with corresponding gene targets targeting begomoviruses genome using different *in silico* algorithm

Through translation suppression and cleavage, a perfect or nearly perfect match between miRNA and target mRNAs controls the expression of post-transcriptional genes. The previously stated particulars were used for forecasting both miRNAs and their targets. Although we predicated 23 miRNAs’ families, the regulation of approx. 820 target transcripts was only observed for those miRNAs which showed its target alignment with our divergent begomoviruse isolates using different *in silico* algorithmic tool. Interestingly, among 23 predicated miRNA families we found 7 miRNA families showing miRNA-target alignment in different genomic region of isolate, in which miR5021 was observed to maximum affinity with begomovirus genes using C-mii tool. Tapirhybrid server identified 18 papaya locus–derived capa-miRNA-target alignment, i.e., capa-miR482, miRNA capa-miR5658, and miRNA capa-miR3441.2 were observed for maximum affinity with begomovirus genes. On the other hand, eight papaya locus-derived capa-miRNA targeting begomoviruses at various locus positions were predicted to have cleavable targets by the psRNATarget algorithm, in which again miR5021 was observed to have maximum affinity with begomovirus genes. Moreover, RNA22 algorithm identified 12 capa-miRNA families in which capa_miR530b, capa_miR529, and capa_miR5021 were observed to have maximum affinity with begomovirus genes (Tables S1–S4).

#### Papaya miRNAs targeting virion-sense ORFs of DNA-A

**Figure 2:**
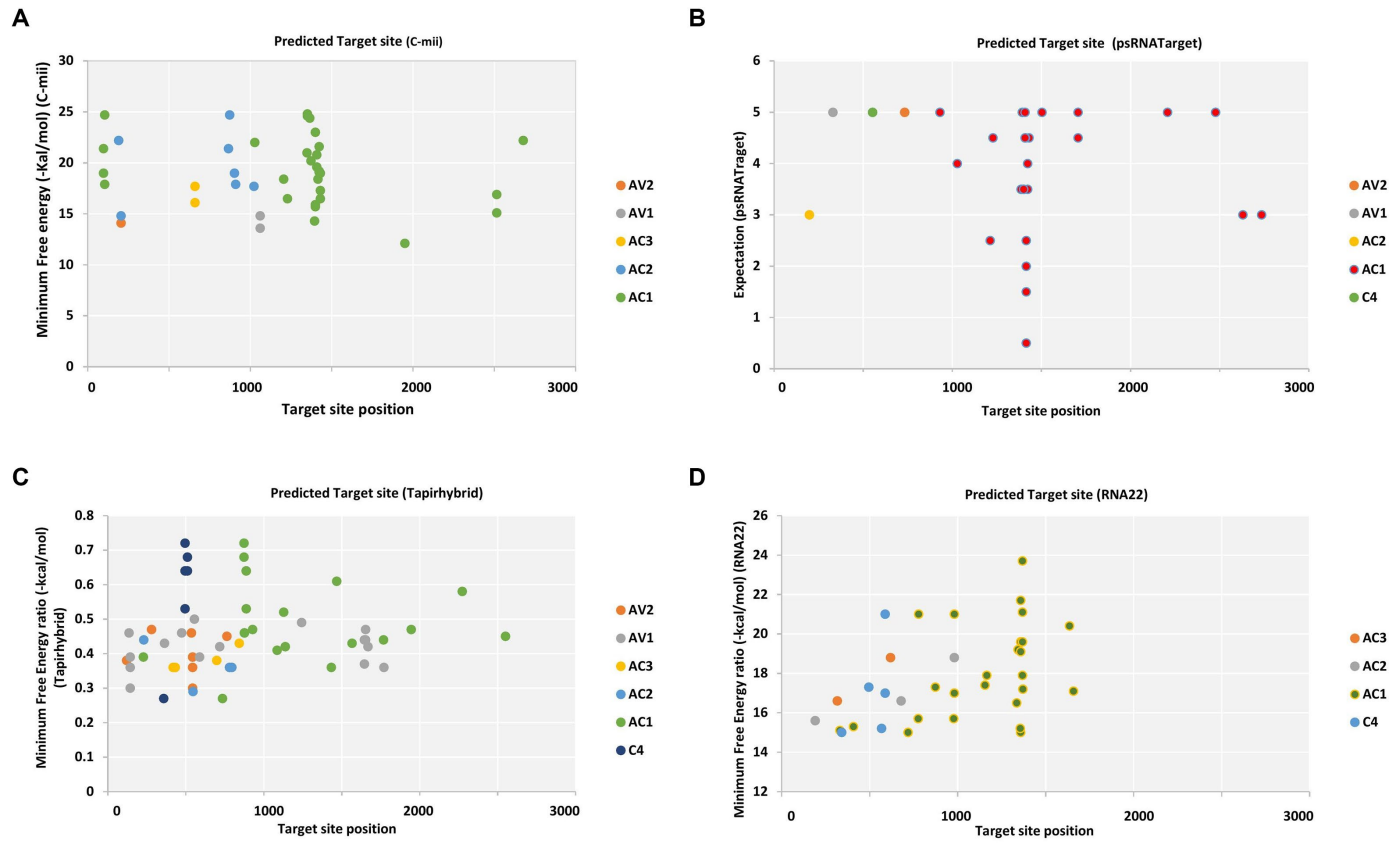
Computational target prediction of papaya miRNAs in the begomovirus genome. **(A)** miRNA-target binding sites obtained from C-mii. **(B)** Predicted miRNA targets obtained by psRNATarget. **(C)** Tapirhybrid-predicted potential hybridization sites. **(D)** The miRNA targets obtained by RNA22. Each viral gene is indicated by a different color, as indicated by the legend shown to the right of the graph, respectively.

Pre-coat protein (pre-CP) and coat protein (CP), which are necessary for the encapsidation of the viral ssDNA genome into virions carried by the whitefly vector and for cell-to-cell migration, are encoded by the begomoviral AV2 ORF and AV1 ORF, respectively (Götz et al., 2012). Different cryptic species or mitotypes of the whitefly vector may transmit at varying rates or with changed competence because of specific mutations in the coat protein (Pan et al., 2020). Among different begomovirus isolates, ORF AV1 and AV2 of CLCuV_AV1_Maharajaganj_RAV were targeted by single predicted miRNA family, i.e., capa-miR837-5p at different locus based on the psRNATarget default algorithm. Furthermore, two miRNAs were predicted by C-mii tool in which AV1 gene of three isolate CLCuV_AV1_Maharajaganj_RAV, CYVMV_AV1_Kahlilabad_RAV, and PaLCuV_AV1_Bilaspur_RAV was targeted by capa-miR5140 and AV2 gene one isolate ToLCNDV_AV2_Mahasamund_RAV was targeted by capa-miR902a-5p at different nucleotide positions. Additionally, the Tapirhybrid algorithm predicted nine papaya locus-derived capa-miRNAs targeting virion-sense ORFs at different locus of isolates ToLCNDV isolate Nautanwa_RAV, Mahasamund_RAV; PaLCuV isolate Gorakhpur_av1, Gorakhpur_av2, Raipur_RAV, Durg_RAV, Bilaspur_RAV, Delhi_RAV, CLCuMuV isolate Bastar_RAV, and CYVMV isolate Kahlilabad_RAV. However, RNA22 algorithm did not identify any predicted capa-miRNA-target pairs working on virion sense (Tables S1–S4; Figure 2).

#### Papaya miRNAs targeting complementary-sense ORFs of DNA-A

Replication-associated proteins are encoded by the complementary strand’s AC1 ORF, transcriptional activator proteins by the AC2 ORF, replication enhancer proteins by the AC3 ORF, and pathogenicity-associated C4 proteins by the AC4 ORF. These viral proteins have a crucial role in the progression of diseases as well as in the replication, transcription, transmission, cell-to-cell migration, repression, and control of genes (Hesketh et al., 2018; Xu et al., 2019).

Several predicted binding sites were identified for the AC1 gene of all the 11 begomovirus isolates and were capa-miR5021, capa-miR529, capa-miR838, capa-miR837-5p, and capa-miR530b target AC1 gene at different locus having overlapping and non-overlapping segment. Furthermore, psRNATarget also identified few predicted capa-miRNAs that can also target AC2 gene of isolate Mahasamund_RAV, Nautanwa_RAV, and C4 gene of isolate Bastar_RAV, but none of the predicted capa-miRNAs are identified associated with AC3 ORF based on psRNATarget default algorithm (Table S2).

The C-mii algorithm identified six predicted miRNA families having overlapping binding sites targeting AC1 gene of isolates CLCuMuV_AC1_Bastar_RAV, CYVMV_AC1_Kahlilabad_RAV, ToLCNDV isolate Mahasamund_RAV, Nautanwa_RAV, PaLCuV isolate Gorakhpur_av1, Delhi_RAV, Durg_RAV, Raipur_RAV, Bilaspur_RAV, Gorakhpur_av2, and CLCuV_AC1_Maharajaganj_RAV, and three miRNAs targeting few AC2 gene of only three isolates, i.e., ToLCNDV isolate Mahasamund_RAV, Nautanwa_RAV, and PaLCuV_AC2_Delhi_RAV. Interestingly, in context to psRNATarget, C-mii algorithm identified one papaya locus-derived capa-miRNA: capa-miR902a-5p to target AC3 gene of PaLCuV isolate Delhi_RAV and Gorakhpur_av2 at common locus 247–264, but none of the predicted capa-miRNAs are identified associated with C4 ORF (Table S1).

The Tapirhybrid algorithm identified eight predicted miRNAs targeting AC1 gene at different locus of PaLCuV isolate Gorakhpur_av1, Durg_RAV, Delhi_RAV, Raipur_RAV Bilaspur_RAV; CYVMV_AC1_Kahlilabad_RAV; ToLCNDV isolate Nautanwa_RAV, Mahasamund_RAV; CLCuV_AC1_Maharajganj_RAV; CLCuMuV_AC1_Bastar_RAV and four predicted miRNAs were found targeting AC2 gene of isolate ToLCNDV_AC2_Mahasamund_RAV, PaLCuV isolate Durg_RAV Gorakhpur_av1, Delhi_RAV, Bilaspur_RAV, and CLCuV_AC2_Maharajganj_RAV at different locus. Similarly, in context to psRNATarget and C-mii algorithm, Tapirhybrid algorithm identified two papaya locus-derived capa-miRNAs for AC3 gene and C4 gene (Table S3).

**Figure 3:**
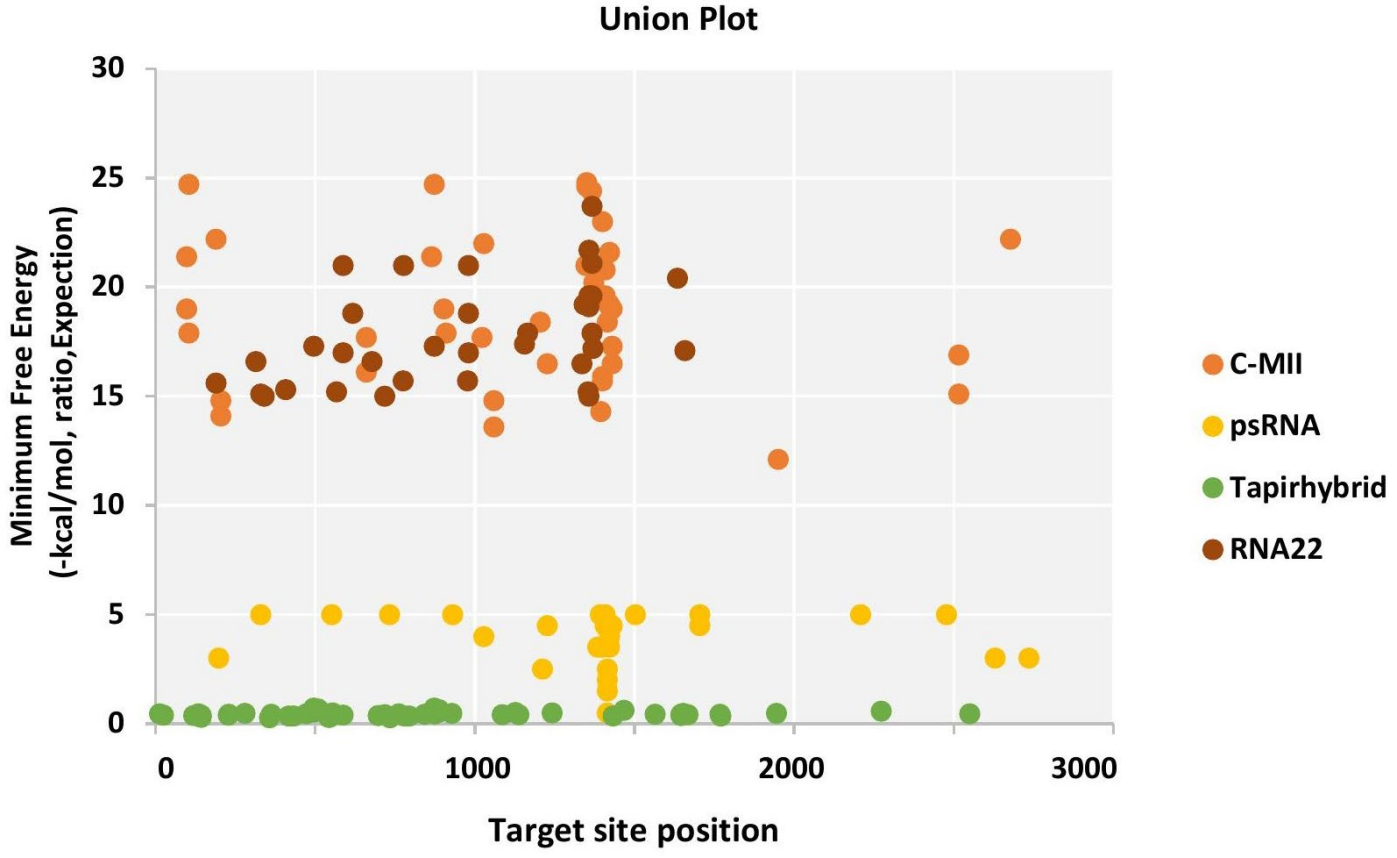
Union plot demonstrating all the predicted papaya miRNA targets in the begomovirus genome, represented as a union from all the four algorithms used.

The RNA22 algorithm identified nine predicted miRNAs targeting AC1 gene, three capa-miRNAs targeting AC2 and C4 gene, and two capa-miRNAs targeting AC3 gene at different nucleotide positions (Table S4; Figure 2). The union plot indicates entire genome binding sites identified by the candidate miRNAs using target prediction tools (Figure 3).

#### Papaya miRNAs targeting associated satellite molecules

Begomoviruses are related with betasatellites that are required for the systemic infection and symptom development and lead to the creation of more pathogenic strains or species with increased disease complexity and to producing outbreaks in new locations (Sharma et al., 2019). Furthermore, begomoviruses have also been linked to a different kind of circular ssDNA satellite; these satellites, formerly known as DNA-1 but currently called alphasatellites, are not known to play a part in the development of symptoms. However, betasatellite encodes a beta C1 ORF, and alphasatellite encodes a Rep ORF; thus, based on psRNATarget default algorithm, three capa-miRNAs: capa-miR838, capa-miR1099, and capa-miR5015b were found to target beta gene of isolate PaLCuB_betaC1_Bilaspur_RAV and CLCuB_betaC1_Mahasamund_RAV, at different locus, but none of them were identified to target Rep gene of alphasatellite.

The Tapirhybrid algorithm identified four capa-miRNAs to target beta C1 genes of betasatellite and Rep gene of alphasatellite. Moreover, based on the C-mii algorithm, none of the papaya locus-derived capa-miRNAs were identified to target beta C1 genes of betasatellite and Rep gene of alphasatellite. However, RNA22 algorithm identified two predicted capa-miRNAs to target beta C1 genes of betasatellite but none for Rep gene of alphasatellite (Tables S1–S4).

#### Papaya miRNAs targeting ORFs of associated DNA-B

The Begomovirus genus comprises both monopartite (containing a single genomic component) and bipartite (containing two genomic components, known as DNA-A and DNA-B) species (Zerbini et al., 2017; Kumar, 2019). The DNA-B of bipartite begomoviruses encodes the movement protein (MP) and nuclear shuttle protein (NSP). MP and NSP work together to facilitate the intra- and intercellular trafficking of viral DNA, and they are necessary for systemic infection to occur. NSP is encoded by ORF BV1 and MP by ORF BC1. Thus, based on psRNATarget default algorithm, the only one capa-miR482 was found to target only BV1 ORF of ToLCNDV_BV1_Durg_RAV isolate at locus 661–680, but none of the predicted capa-miRNAs are identified associated with BC1 gene.

The Tapirhybrid algorithm identified one capa-miRNAs to target BV1 (capa-miR530b) and BC1 (capa-miR1536) gene to target ToLCNDV isolate Durg_RAV. Moreover, based on the C-mii algorithm, none of the papaya locus-derived capa-miRNAs were identified targeting genes of DNA-B. However, RNA22 algorithm identified two predicted papaya locus-derived capa-miRNAs to target genes of DNA-B of ToLCNDV isolate Durg_RAV (Tables S1–S4).

#### Identification of common and unique papaya miRNAs

**Figure 4:**
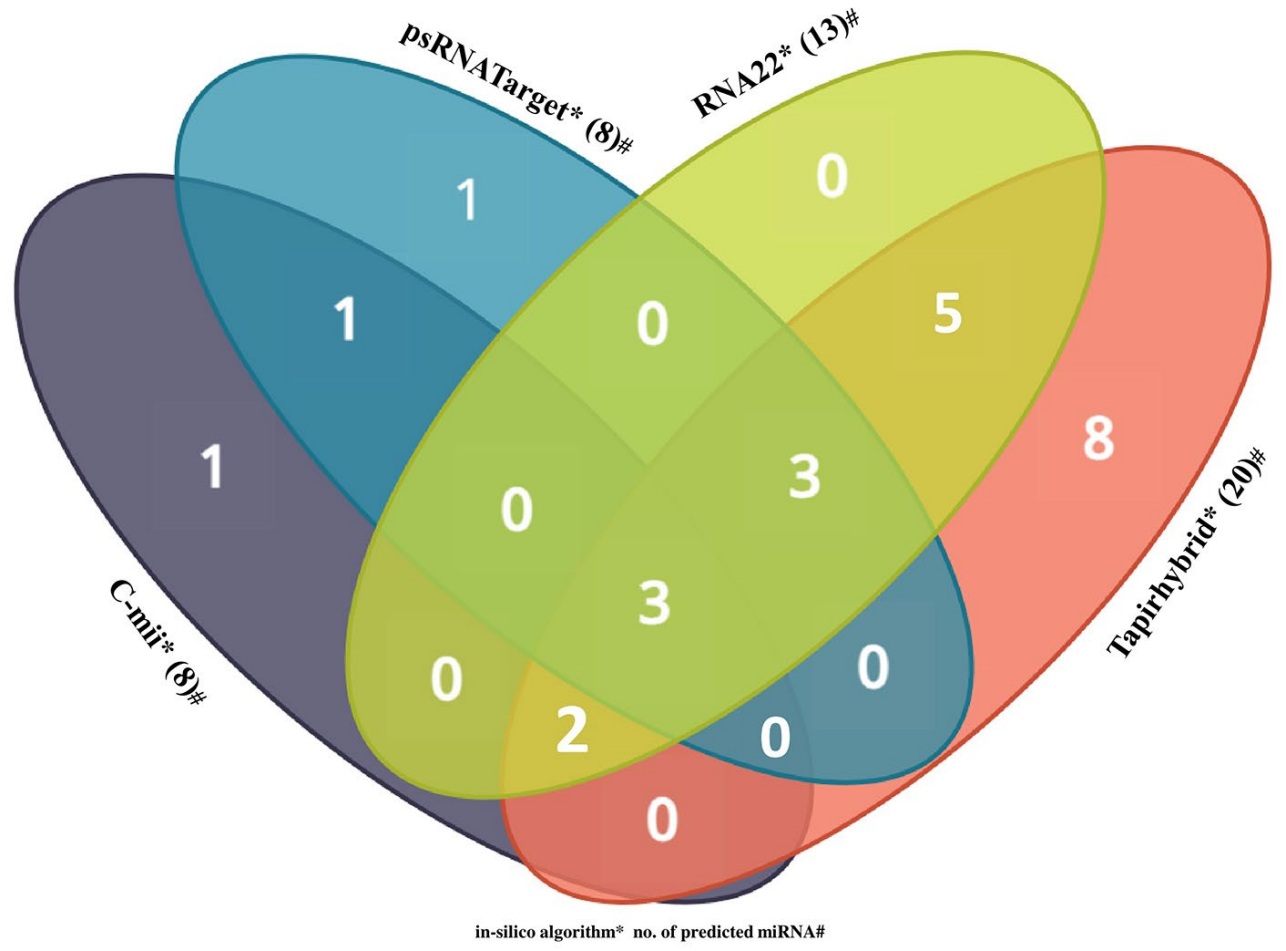
Venn diagram plot of Begomovirus genome targeted by papaya locus-derived capa-miRNA. In total, 48 loci are targeted by papaya miRNAs as predicted from four unique algorithms, i.e., C-mii, psRNATarget, Tapirhybrid, and RNA22 databases.

Among the predicted 48 papaya locus-derived candidate capa-miRNAs, 12 miRNAs: capa-miR5021, capa-miR838, capa-miR529, capa-miR902a-5p, capa-miR530b, capa-miR482, capa-miR837-5p, capa-miR5658, capa-miR5565e, capa-miR5140, capa-miR5015b, and capa-miR1536 were predicted by at least two of the algorithms. Of 12 consensus papaya locus-derived capa-miRNAs, five miRNAs capa-miR529, capa-miR902a-5p, capa-miR482, capa-miR5140, and capa-miR5015b were detected by three algorithms (Tapirhybrid, RNA22, and either by psRNATarget or C-mii). The capa-miR5021, capa-miR838, and capa-miR530b were predicted by all four algorithms (psRNATarget, C-mii, Tapirhybrid, and RNA22), making it the only unique capa-miRNA identified in this study (Table 4; Figure 4).

**Table 4:** Number of papaya miRNAs to target each gene of begomovirus under different algorithms.

Begomovirus genes	C-mii	psRNATarget	Tapirhybrid	RNA22
DNA-A				
*AV1*	miR5140	miR837-5p	miR838; miR529; miR530b; miR5658; miR5565e; miR3441.2; miR414	------
*AV2*	miR902a-5p	miR837-5p	miR1854-3p; miR838; miR5021; miR5658	------
*AC1*	miR837-5p; miR838; miR5021; miR530b; miR902a-5p; miR902a;	miR837-5p; miR838; miR5021; miR529; miR482; miR530b	miR902a-5p; miR482; miR166b; miR166a; miR5672; miR5658; miR776; miR414; miR5015b	miR838; miR5021;miR5658; miR530b; miR529; miR5015b; miR482; miR1536; miR5140
*AC2*	miR838; miR5021; miR902a-5p	miR838	miR5658; miR776; miR5565e; miR3441.2	miR5658; miR902a-5p; miR1854-3p
*AC3*	miR902a-5p	------	miR530b; miR3441.2	miR5658; miR902a-5p
*C4*	------	miR838	miR482; miR414	miR530b; miR5015b; miR838
DNA-B				
*BC1*	------	------	miR1536	miR5565e
*BV1*	------	miR482	miR530b	miR838
Betasatellite				
*betaC1*	------	miR838; miR1099; miR5015b	miR1862g; miR1862f; miR482; miR5658; miR5140	miR5658; miR5015b
Alphasatellite				
*Rep*	------	------	miR530b; miR482; miR5658; miR776	------

**Figure 5:**
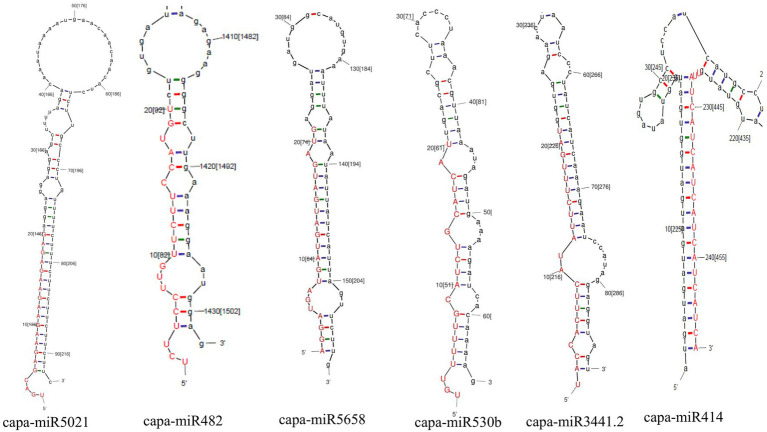
Prediction of secondary structures of stem-loop sequences of papaya miRNAs. Six pre-miRNA secondary structures (precursors of papaya miRNAs) were identified in this study by C-mii algorithm.

Moreover, in this study, based on all four algorithms, capa-miR5021 was the top predicted followed by capa-miR482, capa-miR5658, capa_miR530b, capa-miR3441.2, and capa-miR414 ‘effective’ papaya locus-derived candidate capa-miRNA, harbored potential target binding sites at the consensus nucleotide position, and was predicted to bind and target mostly to AC1 gene of complementary strand and AV1 gene of virion strand of different begomovirus isolates which was associated with replication-associated protein and encapsidation, respectively, during PaLCuD. However, to reduce to complexity in presenting data, the secondary structures were only displayed for six top predicted miRNAs found actively targeting begomovirus AC1, AV1, and betaC1genes and their feature are represented (Table 5; Figure 5).

**Table 5:** Salient parameters of top six miRNAs were determined, showing potential target affinity toward genes of present begomovirus isolate.

Capa-miRNA	Identified algorithm	Target Gene	Variant	MFE range obtained using different algorithm				Salient parameters of precursor miRNAs				
				C-mii (kcal/mol)	psRNATarget (kcal/mol)	Tapirhybrid (kcal/mol)	RNA22 (kcal/mol)					
								Length precursor	Length miRNA	GC content (%)	MFE(kcal/mol)	MFEI(kcal/mol)
capa-miR5021	C-mii, psRNATarget, Tapirhybrid,RNA22	AV2, AC1,AC2	PaLCuV; CLCuMuV; CLCuV; CYVMV; ToLCNDV	−12.1 to − 23.00	−19.1 to − 29.8	−15.3	−15.20 to − 23.70	93 to147	20	32.65 to 44.44	−28.5 to − 140.9	−0.62 to −0.57
capa-miR482	psRNATarget, Tapirhybrid,RNA22	AC1, C4, BV1,	PaLCuV; CLCuMuV; CLCuV; CYVMV ToLCNDV	------	−21.0 to −27.5	−10.7 to −25.9	−17.10 to − 20.40	1,432	20	45.60	−333.7	−0.51
		beta C1, Rep	CLCuB ToLCBDB, PaLCuA; PaLCVSA									
capa-miR5658	Tapirhybrid,RNA22	AV1,AV2, AC1,AC2,AC3	PaLCuV; CYVMV; ToLCNDV	------	------	−10.3 to − 18.3	−15.30 to − 16.60	157	21	37.57	−42.2	−0.71
		beta C1, Rep	ToLCB, PaLCuA,									
capa-miR530b	C-mii, psRNATarget, Tapirhybrid,RNA22	AV1, AC1 AC3, C4 BV1	ToLCNDV PaLCuV CYVMV	−22	−27.7	−13.1 to − 17.6	−15.00 to − 21.00	65	20	35.38	−11.7	−0.50
		Rep	PaLCuA PaLCVSA									
capa-miR3441.2	Tapirhybrid	AV1, AC2, AC3	PaLCuV; CLCuV; CYVMV; ToLCNDV	------	------	−12.6 to − 16.6	------	88	21	36.36	−23.2	−0.61
capa-miR414	Tapirhybrid	AV1, AC1,C4,	PaLCuV; ToLCNDV; CLCuMuV	------	------	−9.9 to − 17	------	246	21	37.39	−67.7	−0.73

### Visualization and analysis of miRNA-mediated gene regulatory network and miRNA-target interaction network

**Figure 6:**
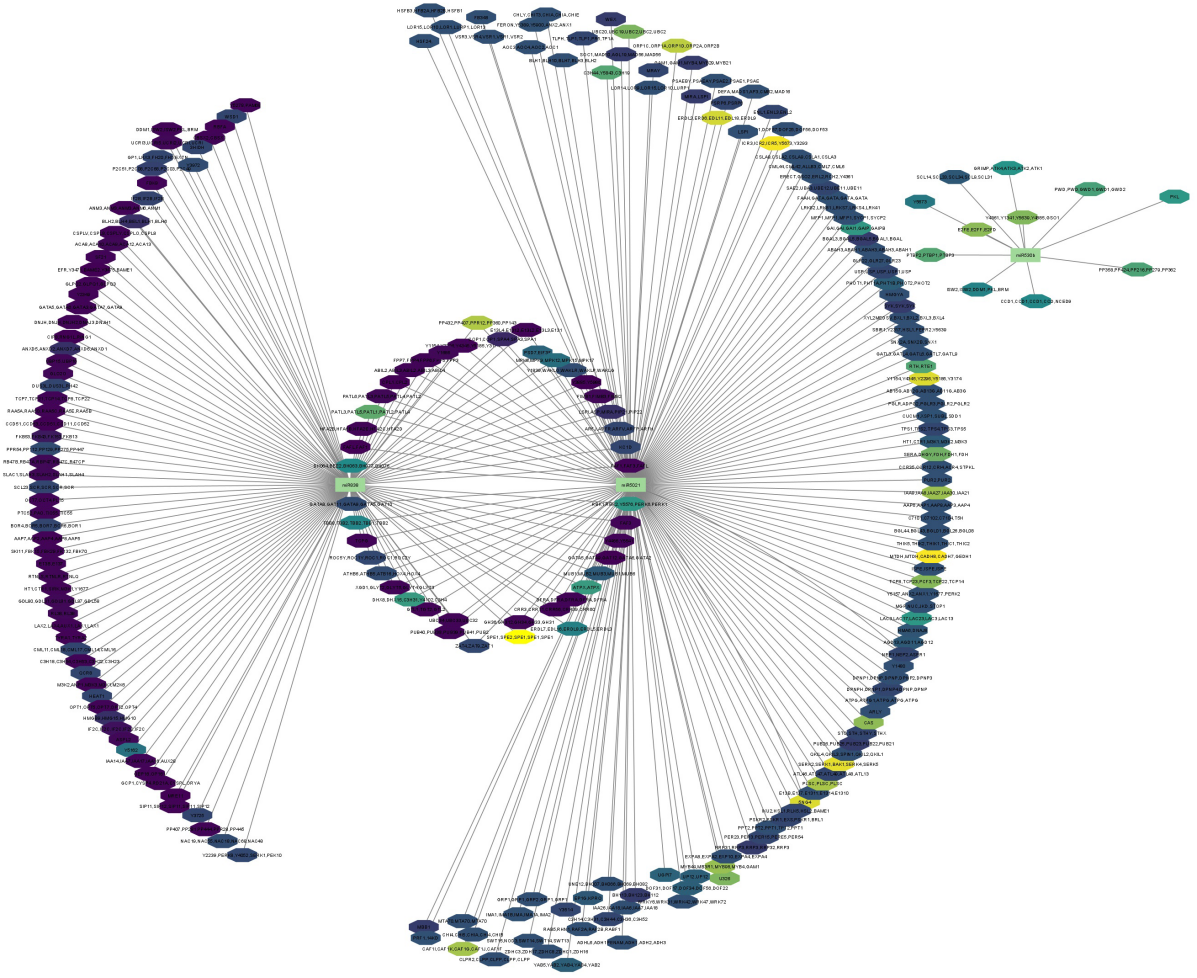
miRNA-mediated gene regulatory network generated using Cytoscape 3.2. software (interaction is shown based on MFE value from light yellow to dark magenta). Different colors of octagon represent the predicted target, and green rectangles represent identified miRNA candidates.

In Figure 6, we present a gene regulatory network illustrating the intricate process of miRNA-mRNA targeting within the plant. Since we predicted large number of miRNA candidates and got huge data on miRNA gene regulation, therefore, we have selectively showcased a subset of examples. Specifically, we highlight three uniquely identified miRNAs to demonstrate the mechanism through which miRNAs modulate gene expression by binding to target mRNAs, resulting in their degradation or translational inhibition (Fabian and Sonenberg, 2012; Ramesh et al., 2014). However, for unique capa-miRNA candidates, i.e.,capa-miR5021, capa-miR838, and capa-miR530b which was predicted by all four algorithms (psRNATarget, C-mii, Tapirhybrid, and RNA22), their miRNA-mediated gene regulatory networks constructed were generated using Cytoscape 3.2 software to reduce visual graphical complexity and allow improved readability (Figure 6). Based on the MFE value, the miRNA-mediated gene regulatory network is continuously mapped from light yellow to dark magenta, that is, the greater the MFE value, the darker the color and the greater the interaction magnitude, and vice versa.

**Figure 7:**
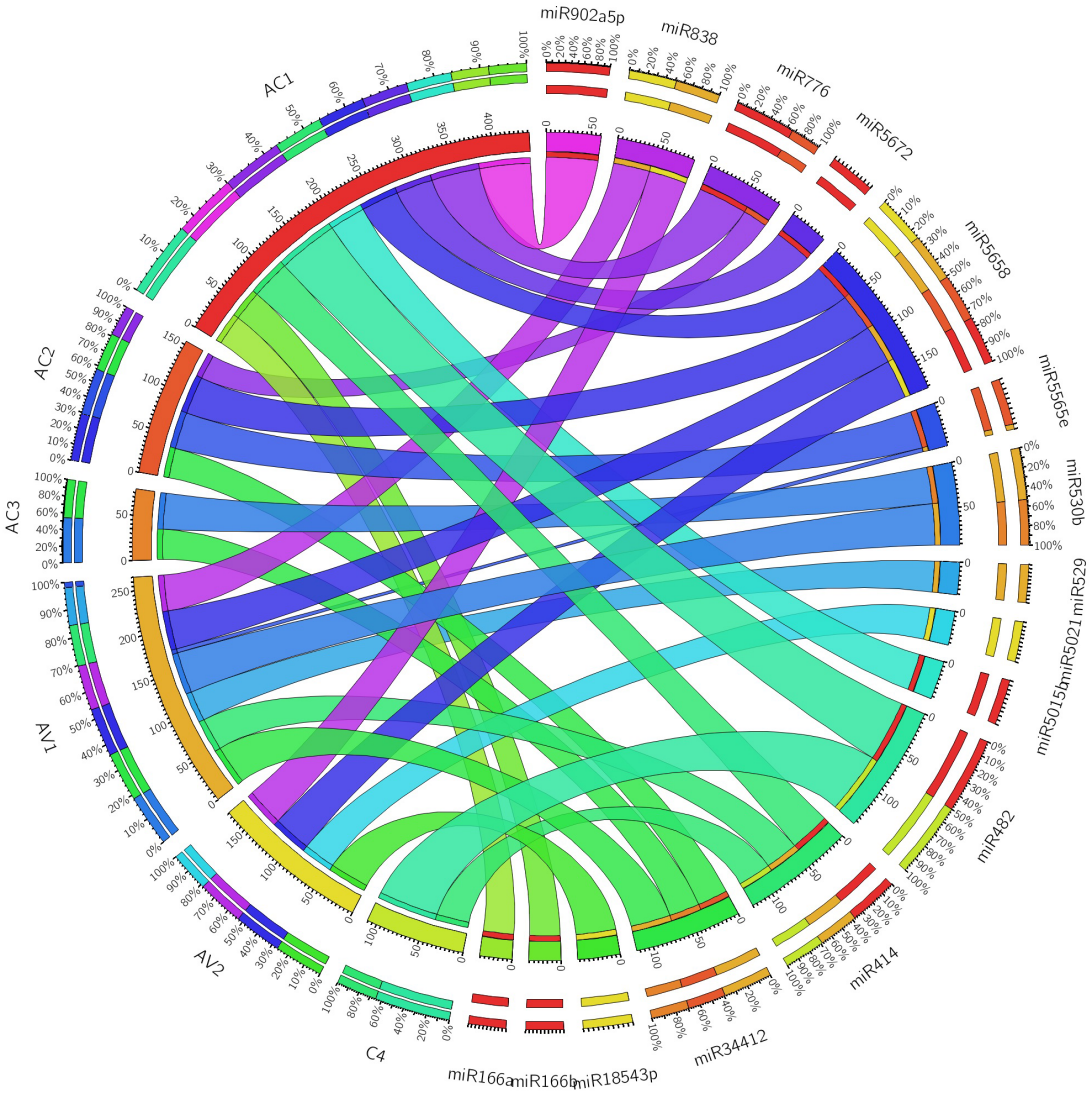
Circos plot representing begomovirus gene interaction predicted to be targeted by capa-miRNAs. The colored connection lines represent target interactions between the papaya locus-derived capa-miRNAs and viral genes based on Tapirhybrid algorithm.

Additionally, we developed a Circos plot to integrate biological data from papaya miRNAs and their predicted begomovirus genomic target genes (ORFs) to provide a thorough visualization of host–virus interaction. We only used a subset of papaya miRNAs and their targets that came from Tapirhybrid analysis for the miRNA-target interaction network. The Tapirhybrid algorithm’s output identified the capa-miRNA predicted to target every gene known to be associated with begomovirus. Other algorithms, however, were unable to forecast that all six ORFs of the begomovirus genomic DNA-A would be targeted (Figure 7). According to the findings, using consensual miRNAs, biological data visualization of candidate miRNAs from papaya with begomovirus-encoded ORFs identifies reliable information about desirable preferred targets.

#### Evaluation of free energy (ΔG) of mRNA-miRNA interactions

By computing the free energies (ΔG) of the miRNA/target duplexes for duplexes for begomovirus isolate and associated satellite according to psRNATarget, the predicted papaya locus-derived capa-miRNA-mRNA target pairs were assessed and verified (Table 6). The free energy change (ΔG) is a measure of the thermodynamic stability of the interaction. A negative ΔG indicates that the interaction is energetically favorable and more likely to occur spontaneously. A lower (more negative) ΔG suggests a stronger and more stable interaction (Bernhart et al., 2006). A complete complementary between miRNA-mRNA sequences leads to cleavage of the targeted mRNA, while partial complementary typically decreases gene expression by suppressing translation of target mRNA (Mishra et al., 2020).

**Table 6:** Free energy (Δ*G*) of the consensus papaya locus-derived capa-miRNA-mRNA target pairs was estimated based on psRNATarget.

miRNA	Target	Gene	capa-miRNA- target alignment			ΔG Duplex (Kcal/mol)	ΔG Binding (Kcal/mol)
capa-miR837-5p	PaLCuV	AC1	CUUUGUUUUUUUUUUUUUUCU	:.: .::.:.:::::.:::	UGGACGAAGAGAAAAAUAACG	−11.10	−11.63
capa-miR5021	PaLCuV	AC1	UGACGAGAAGAAGAAGAGAG	.:::::::::::.::::	UUAUCUUCUUCUUUUAAUCA	−16.70	−17.31
capa-miR529	PaLCuV	AC1	GAAGAAG-AGAGAAGGAAGAA	:::::.:::::::::::::	UUCUCCUUUCUCUUCUUCUUC	−21.90	−23.76
capa-miR482	PaLCuV	AC1	UCUUCCUUGUUCUUCCAUGU	:::::::::.:::::	UGAUAGUAGAACAGGAAAGA	−12.30	−13.32
capa-miR5021	CLCuMuV	AC1	UGACGAGAAGAAGAAGAGAG	:.::::::.:::::::.	AUUUCUCCUUUUUCUCGUUC	−20.00	−20.48
capa-miR529	CLCuMuV	AC1	GAAGAA-GAGAGAAGGAAGAA	:::::.::.::::::::::	UUCUCCUUUUUCUCGUUCUUC	−19.10	−20.21
capa-miR838	CLCuMuV	AC1	UCUUCUUCUUCUUCUUCUUCA	:::::: .::::.::.::	GGAAGAUCAGCAAGAGGAGGA	−17.40	−18.52
capa-miR837-5p	CLCuV	AV1	CUUUGUUUUUUUUUUUUUUCU	::::::::.:.::: .:	ACAAACAAAAGGAGGACAUGG	−8.30	−9.48
capa-miR837-5p	CLCuV	AV2	CUUUGUUUUUUUUUUUUUUCU	::::::::.:.::: .:	ACAAACAAAAGGAGGACAUGG	−8.30	−9.48
capa-miR5021	CLCuV	AC1	AAAGAAGAAGAAGAAGAAGA	::.:: .:::::::::::	CCCUUUUUAUCUUCUUCUUU	−17.20	−17.83
capa-miR529	CLCuV	AC1	GAAGAAGA-GAGAAGGAAGAA	::::::::.: .::::::::	UUCUCCCUUUUUAUCUUCUUC	−19.20	−19.91
capa-miR5021	CYVMV	AC1	UGACGAGAAGAAGAAGAGAG	:::::::::::.::::	UCCUCUUCUUCUUUUGAUCA	−20.60	−20.80
capa-miR529	CYVMV	AC1	GAAGAAGA-GAGAAGGAAGAA	:::::.::.::::::::::	UUCUCCUUUUUCCUCUUCUUC	−19.70	−21.26
capa-miR837-5p	ToLCNDV	AC1	CUUUGUUUUUUUUUUUUUUCU	:.: .::.::::::: .::	UGGACGAAGAAAAAAAUGCAG	−9.90	−11.26
capa-miR838	ToLCNDV	AC1	UCUUCUUCUUCUUCUUCUUCA	:::::::::::::::: .:	CGCAGAAGAAGAAGAAGAGCA	−22.90	−23.39
capa-miR838	ToLCNDV	AC2	UCUUCUUCUUCUUCUUCUUCA	:::::::::::::::: .:	CGCAGAAGAAGAAGAAGAGCA	−22.90	−23.39
capa-miR530b	ToLCNDV	AC1	UGUUUUUGCAUCUGCAUCAU	::::::::::::::.: .	ACGAUGCAUAUGCAAAGGCG	−22.20	−18.50
capa-miR482	ToLCNDV	BV1	UCUUCCUUGUUCUUCCAUGU	::.::::.::.::::::	ACGUUCAAGGACGAAGAAGA	−13.80	−13.17
capa-miR838	PaLCuB	betaC1	UCUUCUUCUUCUUCUUCUUCA	::::::::.:::.::	ACAACAAGAAGGGGAUGGAGU	−15.70	−16.41
capa-miR5015b	PaLCuB	betaC1	UCUUUUGUUGUUGUUGUUGUU	::::::::::::::.: .	AAAUACAACAACAAGAAGGGG	−17.80	−18.67
capa-miR1099	CLCuB	betaC1	UUUAGCAAUGGUGAAUAUGUC	::::::::::::::::	CACAAAGUCACCAUCGCUAAU	−17.70	−17.53

ΔG represents minimum free energy of duplex formation.

## Discussion

Originally, the papaya plant was flourished in Central America and Southern Mexico. It is currently grown in India and many other nations, including South Africa and Sri Lanka. The states that grow papayas the most are Andhra Pradesh, Madhya Pradesh, Gujarat, and Karnataka. Over the years, the papaya crop has faced a number of biotic and abiotic challenges. The most well-known of these is PaLCuD, which is largely to blame for the significant decline in papaya production. PaLCuD was first described by Thomas and Krishnaswamy in 1939, and the causative pathogen was subsequently discovered (Saxena et al., 1998). In all nations that grow papaya plants and where whiteflies are a common major or minor pest, PaLCuD is a terrifying presence.

Over the past 20 years, the disease has spread to India and neighboring regions, where it is triggered by a few monopartite and a few bipartite begomoviruses that can infect different varieties of papaya, while specific varieties that are more susceptible to begomoviruses are not mentioned. Therefore, it is important to note that the disease can impact plant growth, fruit size, quality, and quantity in papaya plants. The exact production loss of *Carica papaya* each year due to viral diseases is not specified in the available literature. However, it is mentioned that papaya is severely damaged by diseases such as papaya ringspot virus and papaya leaf curl virus, which can cause up to 40–100% crop losses in some regions (Alabi et al., 2016; Alcala-Briseno et al., 2020; Srivastava et al., 2022a). Begomoviruses, including papaya leaf curl virus, can significantly impact papaya production. To mitigate these losses, preventive measures such as disease-resistant varieties and proper crop management practices are recommended.

Centered on *in silico* standards, numerous investigations have looked into possible endogenous host–plant mature microRNAs that could target plant viruses (Ashraf et al., 2021, 2022). As important endogenous biomolecules for controlling gene expression, miRNAs have developed over time. However, miRNAs targeting viral genes in *C. papaya* is not yet studied. Further research in this area may provide insights into the potential involvement of miRNAs in targeting viral genes, including those of viruses affecting *C. papaya*.

To predict the most efficient miRNA-target binding sites and the precise interactions with the AV1, AV2, AC1, AC2, AC3, C4 gene of DNA-A; BC1, BV1 genes of DNA-B; betaC1 gene of betasatellite and Rep gene of alphasatellite, mature papaya miRNAs were hybridized *in silico* with the 11 distinct begomovirus isolates in the present investigation. Four computational algorithms, i.e., c-mii, psRNATarget, RNA22, and Tapirhybrid, were used.

However, in the current study, a novel set of papaya microRNAs (miRNAs) has been successfully identified through the analysis of papaya leaf transcriptome data. These miRNAs are newly discovered in our investigation and have not been previously reported in any of the existing miRNA databases. The utilization of transcriptome data provided a valuable resource for expanding our understanding of the papaya genome’s regulatory landscape.

Using *in silico* tools and techniques, we found mature candidates of papaya locus-derived capa-miRNAs, which were predicted to have effective target binding sites in the genomes of different begomoviruses isolates. In total, 48 loci are targeted by papaya miRNAs as predicted from four algorithms, i.e., C-mii, psRNATarget, Tapirhybrid, and RNA22 databases. From 48 targeting loci, we identified the Common and Unique papaya miRNAs based on four algorithms. We have also found 12 capa-miRNAs, predicted by at least two of the algorithms. From 12 miRNAs, five miRNAs were detected by three algorithms (Tapirhybrid, RNA22, and either by psRNATarget or C-mii). Based on all four algorithms, capa-miR5021 was the top predicted followed by capa-miR482, capa-miR5658, capa_miR530b, capa-miR3441.2, and capa-miR414 ‘effective’ papaya locus-derived candidate capa-miRNA and harbored potential target binding sites at the consensus nucleotide position. The secondary structures were only displayed for these six top predicted miRNAs which were found actively targeting begomovirus AC1, AV1, and betaC1 gene of identified begomovirus isolate. A common parameter for miRNA prediction and evolutionary inferences is the MFE (Thody et al., 2020). For the chosen miRNA-mRNA target pairs, the maximum MFE range and other silent features are discussed.

Consequently, for three identified unique capa-miRNA, miRNA-mediated gene regulatory network was generated to explain the role of gene expression. Moreover, among four algorithm, the Tapirhybrid algorithm’s output identified the capa-miRNA predicted to target every gene known to be associated with begomovirus and were visualized for host–virus interaction.

In context to free energy measurements that showcase the fluctuating properties of miRNAs and their intended binding, a set of paired criteria has been developed for predicted capa-miRNAs that, when expressed in *C. papaya*, have a strong potential for RNA interference (RNAi) and gene silencing of different begomovirus genomes. Between geminiviruses, the begomoviral CP (AV1) and Rep (AC1) protein is highly conserved (Ruhel and Chakraborty, 2019). The papaya consensus capa-miRNA (Table 6) has been entailed in the present investigation’s *in silico* analyses as highly targeting the distinct begomovirus isolate’s encoding region. However, we have found potential capa-miRNA to target gene of interest, i.e., AV1 gene—capsid protein—encapsidation of ssDNA; whitefly-mediated transmission; AC1 gene—replication-associated protein—initiating and regulating the replication process; betaC1—suppresses host defenses and influences symptom development.

The free energy (Δ*G*) of the consensus papaya locus-derived capa-miRNA-mRNA target pairs was estimated based on psRNATarget which indicates that the interaction is energetically favorable for miRNA-mRNA duplexes that are predicted to represent ‘true targets’ (Riffo-Campos et al., 1987) and probably going to show strong cleavage and/or translation inhibition and hybridization (annealing/binding) in the seed region (Peterson et al., 2014). The papaya encoded miRNA-mRNA duplex exhibited a low MFE value, which serves as one measure of controlling for false positive results. When combined with the results of the ‘four algorithms’ approach to filter false positive targets, the psRNATarget approach was expected to result in a highly sensitive and specific *in silico* strategy for predicting ‘true’ interactors (Oliveira et al., 2017; Quillet et al., 2021) among the miRNA-mRNA target pairs.

Research on the relationship among begomovirus-associated papaya leaf curl diseases and capa-miRNAs derived from the papaya locus is essential as it represents the first steps toward developing miRNA-based anti-plant viral treatments. The outcomes of this research hold the promise of uncovering novel regulatory mechanisms involved in the host–pathogen interaction between papaya-miRNA and begomoviruses genes. Moreover, the identified miRNAs may serve as potential candidates for the development of targeted strategies to control PaLCuD, thereby contributing to the sustainable management of papaya crops and ensuring food security in regions susceptible to begomovirus infections.

To develop begomovirus resistance in papaya plants, future research will concentrate on validating this exciting capa-miRNA derived from the papaya locus to show whether these predicted miRNAs could make the plants resistant to diverse begomovirus species. This study will also involve assessing the significance of the predicted consensus miRNAs obtained from the papaya locus in begomovirus replication to reduced reliance on chemical interventions and increased crop resilience that can enhance long-term productivity contributing to sustainable agriculture. To create a successful bioinformatics workflow for predicting viral genome silencing and move forward with the development of genetically engineered papaya crops with tolerance or resistance that use host plant genome-encoded miRNAs to combat the mono/bipartite begomovirus, this study assessed 48 papaya miRNAs that had been *in vitro* validated for their potential to interact with annotated targets encoded by the begomovirus genome. MiRNAs have revolutionized our understanding of gene regulation, and their discovery has opened new avenues for research into various biological processes and potential therapeutic interventions. Researchers are still actively investigating the roles and functions of miRNAs in different organisms and contexts, and the field of miRNA research continues to evolve.

## Conclusion

An emergent begomoviral pathogen linked to the ongoing PaLCuD crisis, and papaya leaf curl disease reduces yield and vigor in all cultivars of papaya currently grown in the region. It affects papaya crops throughout India.

Here, mature candidates of papaya locus-derived capa-miRNAs were predicted to have effective target binding sites in the genomes of different begomoviruses using *in silico* tools and techniques. The viral genome’s maximum predicted hybridization sites were found in the 48 papaya miRNAs that were studied. Among them, capa-miR5021 followed by capa-miR482, capa-miR5658, capa-miR3441.2, capa-miR414, and capa_miR530b was identified as a highly promising, naturally occurring biomolecule with potential to modulate virus infection and reduce damage to the plant host.

Research has indicated that the expression of amiRNA in genetically modified crops effectively reduces plant virus infection, for both DNA and RNA viral genomes (Yang et al., 2021; Yasir et al., 2022). High specificity for base-pairing with the target gene was demonstrated by the amiRNA construct, which is anticipated to reduce harmful off-target effects and promote stable and dependable security in ensuing generations of progeny plants (Kampmann et al., 2015). A novel method for understanding a variety of cellular processes and forecasting host-derived, virus-specific parameters is RNAi-screening (Martin et al., 2018).

A crucial initial phase in assessing host–virus relationships in light of environmental stresses, such as virus infection, is the identification, analysis, and validation of mature miRNAs in papaya. This process is aided by regulatory network evaluation of the host plant (Rogans and Rey, 2016). To identify relevant virus-specific targets, the predicted engagement of the capa-miRNAs derived from the papaya locus in begomoviral interactions associated with papaya host plants was further analyzed. The aim is to achieve amiRNA-mediated resistance to the newly emerging begomoviruses in Indian papaya crops by expressing the most promising *in silico* papaya locus-derived capa-miRNAs in transgenic papaya plants.

To lessen the effects of PaLCuD, future research will concentrate on creating papaya plants immune to begomovirus. The current findings highlight the value of using a combined *in silico*-screen-molecular approach to create amiRNA treatment options for the management of PaLCuD and other emerging begomoviral infections that pose a global threat to food and fiber crops.

## Data availability statement

The datasets presented in this study can be found in online repositories. The names of the repository/repositories and accession number(s) can be found in the article/Supplementary material.

## Author contributions

AS: Data curation, Formal analysis, Software, Writing – original draft. VP: Data curation, Formal analysis, Software, Writing – original draft. NS: Formal analysis, Validation, Writing – original draft. AM: Formal analysis, Validation, Writing – review & editing. MS: Conceptualization, Writing – review & editing. RG: Conceptualization, Writing – review & editing.

## Footnotes

^1^ https://www.ncbi.nlm.nih.gov/genome/annotation_euk/Carica_papaya/100/

^2^ usegalaxy.org

^3^ http://ncbi.nlm.nih.gov

^4^http://www.sanger.ac.uk/Software/Rfam/ and http://rfam.wustl.edu/

^5^ http://rna.tbi.univie.ac.at/cgi-bin/RNAfold.cgi

^6^ http://mkweb.bcgsc.ca/tableviewer/visualize/
