# Supplementary material for: In silico identification of papaya genome-encoded microRNAs to target begomovirus genes in papaya leaf curl disease
Source: Front Microbiol. 2024 Mar 21;15:1340275. doi: 10.3389/fmicb.2024.1340275 (PMC11008722; doi:10.3389/fmicb.2024.1340275)
Supplement: Supplementary file 1 [file Table_1.pdf]

Table S1: identification of miRNA-mRNA target predicted to bind the genes of begomovirus isolates using C-mii tool

miRNA_Acc.	Target_Acc.	MFE (kcal/mol)	Locus
capa-miR837-5p	CLCuMuV_AC1_Bastar_RAV	-14.3	538-558
capa-miR838	CLCuMuV_AC1_Bastar_RAV	-20.2	529-549
capa-miR838	CYVMV_AC1_Kahlilabad_RAV	-24.6	520-540
capa-miR838	ToLCNDV_AC1_Mahasamund_RAV	-22.2	1071-1091
capa-miR838	PaLCuV_AC1_Gorakhpur_av1	-21	519-539
capa-miR838	PaLCuV_AC1_Delhi_RAV	-24.4	526-546
capa-miR838	PaLCuV_AC1_Durg_RAV	-24.8	520-540
capa-miR838	ToLCNDV_AC2_Mahasamund_RAV	-22.2	56-76
capa-miR838	PaLCuV_AC1_Raipur_RAV	-24.8	520-540
capa-miR5021	CLCuMuV_AC1_Bastar_RAV	-15.7	541-560
capa-miR5021	CLCuV_AC1_Maharajaganj_RAV	-15.9	541-560
capa-miR5021	CYVMV_AC1_Kahlilabad_RAV	-19.6	544-563
capa-miR5021	CYVMV_AC1_Kahlilabad_RAV	-17.3	553-572
capa-miR5021	PaLCuV_AC1_Bilaspur_RAV	-18.4	463-482
capa-miR5021	PaLCuV_AC1_Bilaspur_RAV	-16.5	472-491
capa-miR5021	PaLCuV_AC1_Delhi_RAV	-20.8	544-563
capa-miR5021	PaLCuV_AC1_Delhi_RAV	-19	553-572
capa-miR5021	PaLCuV_AC1_Delhi_RAV	-21.6	550-569
capa-miR5021	PaLCuV_AC1_Durg_RAV	-23	541-560
capa-miR5021	PaLCuV_AC1_Durg_RAV	-19.1	550-569
capa-miR5021	PaLCuV_AC1_Durg_RAV	-19.2	550-569
capa-miR5021	PaLCuV_AC1_Gorakhpur_av1	-12.1	761-780
capa-miR5021	PaLCuV_AC1_Gorakhpur_av2	-18.4	547-566
capa-miR5021	PaLCuV_AC1_Gorakhpur_av2	-16.5	553-572
capa-miR5021	PaLCuV_AC1_Raipur_RAV	-23	541-560
capa-miR5021	PaLCuV_AC1_Raipur_RAV	-19.1	550-569
capa-miR5021	PaLCuV_AC1_Raipur_RAV	-19.2	550-569
capa-miR5021	ToLCNDV_AC1_Mahasamund_RAV	-21.4	20-39
capa-miR5021	ToLCNDV_AC1_Mahasamund_RAV	-24.7	23-42
capa-miR5021	ToLCNDV_AC2_Mahasamund_RAV	-24.7	330-349
capa-miR5021	ToLCNDV_AC2_Nautanwa_RAV	-19	342-361
capa-miR5021	ToLCNDV_AC2_Nautanwa_RAV	-17.9	345-364
capa-miR5021	ToLCNDV_AC1_Nautanwa_RAV	-19	20-39
capa-miR5021	ToLCNDV_AC1_Nautanwa_RAV	-17.9	23-42
capa-miR5021	ToLCNDV_AC2_Mahasamund_RAV	-21.4	327-346
capa-miR5140	CLCuV_AV1_Maharajaganj_RAV	-14.8	407-424
capa-miR5140	CYVMV_AV1_Kahlilabad_RAV	-14.8	407-424

capa-miR5140	PaLCuV_AV1_Bilaspur_RAV	-13.6	407-424
capa-miR902a-5p	PaLCuV_AC1_Gorakhpur_av2	-16.9	989-1006
capa-miR902a-5p	PaLCuV_AC2_Delhi_RAV	-17.7	392-409
capa-miR902a-5p	PaLCuV_AC3_Delhi_RAV	-17.7	247-264
capa-miR902a-5p	PaLCuV_AC3_Gorakhpur_av2	-16.1	247-264
capa-miR902a-5p	ToLCNDV_AV2_Mahasamund_RAV	-14.1	65-82
capa-miR902a-5p	ToLCNDV_AC2_Nautanwa_RAV	-14.8	65-82
capa-miR902a	PaLCuV_AC1_Durg_RAV	-15.1	989-1006
capa-miR530b	ToLCNDV_AC1_Nautanwa_RAV	-22	392-411

Table S2: Identification of potential binding sites of papaya locus-derived capa-miRNAs predicted to bind the genes of begomovirus isolates using psRNATarget database.

miRNA_Acc.	Target_Acc.	Expectation	Target Binding Locus Position	capa-miRNA- target alignment			Inhibition	MFE (kcal/mol)
capa-miR837-5p	CLCuV_AV1_Maharajganj_RAV	5	112-132	CUUUGUUUUUUUUUUUUUUCU	: ::: ::::..:..::: .:	ACAAACAAAAGGAGGACAUGG	Cleavage	-14.0 kcal/mol
capa-miR837-5p	CLCuV_AV2_Maharajganj_RAV	5	272-293	CUUUGUUUUUUUUUUUUUUCU	: ::: ::::..:..::: .:	ACAAACAAAAGGAGGACAUGG	Cleavage	-14.0 kcal/mol
capa-miR837-5p	ToLCNDV_AC1_Mahasamund_RAV	5	971-991	CUUUGUUUUUUUUUUUUUUCU	:.: .::.:::::::.. ::	UGGACGAAGAAAAAAAUGCAG	Cleavage	-22.0 kcal/mol
capa-miR837-5p	PaLCuV_AC1_Bilaspur_RAV	5	863-883	CUUUGUUUUUUUUUUUUUUCU	:.: .::.:.:::::.:: :	UGGACGAAGAGAAAAAUAACG	Cleavage	-16.1 kcal/mol
capa-miR5021	PaLCuV_AC1_Durg_RAV	0.5	547-566	AAAGAAGAAGAAGAAGAAGA	:::.::::::::::::::::	CCUUUUUCUUCUUCUUCUUU	Cleavage	-29.8 kcal/mol
capa-miR5021	PaLCuV_AC1_Durg_RAV	4	550-569	UGACGAGAAGAAGAAGAGAG	.:.:::::::::::. : :	UUUUCUUCUUCUUCUUUUGA	Cleavage	-25.3 kcal/mol
capa-miR5021	PaLCuV_AC1_Raipur_RAV	0.5	547-566	AAAGAAGAAGAAGAAGAAGA	:::.::::::::::::::::	CCUUUUUCUUCUUCUUCUUU	Cleavage	-29.8 kcal/mol
capa-miR5021	PaLCuV_AC1_Raipur_RAV	4	550-569	UGACGAGAAGAAGAAGAGAG	.:.:::::::::::. : :	UUUUCUUCUUCUUCUUUUGA	Cleavage	-25.3 kcal/mol
capa-miR5021	PaLCuV_AC1_Delhi_RAV	1.5	547-566	AAAGAAGAAGAAGAAGAAGA	:::. :::::::::::::	CCUUUCUCUUCUUCUUCUUU	Cleavage	-26.6 kcal/mol
capa-miR5021	PaLCuV_AC1_Delhi_RAV	3.5	550-569	UGACGAGAAGAAGAAGAGAG	.:::::::::::::. : :	UUCUCUUCUUCUUCUUUUGA	Cleavage	-28.7 kcal/mol
capa-miR5021	CYVMV_AC1_Kahlilabad_RAV	2	547-566	AAAGAAGAAGAAGAAGAAGA	:::.::: :::::::::::	CCUUUUUCCUCUUCUUCUUU	Cleavage	-25.7 kcal/mol
capa-miR5021	CYVMV_AC1_Kahlilabad_RAV	4.5	553-572	UGACGAGAAGAAGAAGAGAG	:::::::::::.: :::	UCCUCUUCUUCUUUUGAUCA	Cleavage	-24.9 kcal/mol
capa-miR5021	PaLCuV_AC1_Gorakhpur_av2	2.5	547-566	AAAGAAGAAGAAGAAGAAGA	:::.::. ::::::::::	CCUUUUUUAUCUUCUUCUUU	Cleavage	-25.7 kcal/mol
capa-miR5021	PaLCuV_AC1_Gorakhpur_av2	4.5	553-572	UGACGAGAAGAAGAAGAGAG	.: ::::::::::.: :::	UUAUCUUCUUCUUUUAAUCA	Cleavage	-22.8 kcal/mol
capa-miR5021	PaLCuV_AC1_Bilaspur_RAV	2.5	466-485	AAAGAAGAAGAAGAAGAAGA	:::.::. ::::::::::	CCUUUUUUAUCUUCUUCUUU	Cleavage	-24.0 kcal/mol
capa-miR5021	PaLCuV_AC1_Bilaspur_RAV	4.5	472-491	UGACGAGAAGAAGAAGAGAG	.: ::::::::::.: :::	UUAUCUUCUUCUUUUAAUCA	Cleavage	-22.8 kcal/mol
capa-miR5021	CLCuMuV_AC1_Bastar_RAV	3.5	541-560	AAAGAAGAAGAAGAAGAAGA	:::.:::: ::::::::	CCUUUUUCUCGUUCUUCUUU	Translation	-22.5 kcal/mol
capa-miR5021	CLCuMuV_AC1_Bastar_RAV	3.5	535-554	UGACGAGAAGAAGAAGAGAG	:.::: :::.:::::::.	AUUUCUCCUUUUUCUCGUUC	Cleavage	-25.3 kcal/mol
capa-miR5021	PaLCuV_AC1_Gorakhpur_av1	4.5	545-564	AAAGAAGAAGAAGAAGAAGA	.:::: :::.:::::: :	GUUUCUCCUUUUUCUUCAUC	Cleavage	-21.4 kcal/mol
capa-miR5021	PaLCuV_AC1_Gorakhpur_av1	4.5	545-564	UAUGUAGAAGAAGAAGAAGA	.:::: :::.:::: :::	GUUUCUCCUUUUUCUUCAUC	Cleavage	-19.1 kcal/mol
capa-miR5021	CLCuV_AC1_Maharajganj_RAV	3.5	541-560	AAAGAAGAAGAAGAAGAAGA	: :.::. ::::::::::	CCCUUUUUAUCUUCUUCUUU	Cleavage	-21.9 kcal/mol
capa-miR529	CLCuV_AC1_Maharajganj_RAV	5	537-557	GAAGAAGA-GAGAAGGAAGAA	:::: ::::.:. ::::::::	UUCUCCCUUUUUAUCUUCUUC	Cleavage	-25.2 kcal/mol
capa-miR529	PaLCuV_AC1_Durg_RAV	5	543-563	GAAGAAG-AGAGAAGGAAGAA	:::: :.::.::: :::::::	UUCUCCUUUUUCUUCUUCUUC	Cleavage	-25.3 kcal/mol
capa-miR529	PaLCuV_AC1_Raipur_RAV	5	543-563	GAAGAAG-AGAGAAGGAAGAA	:::: :.::.::: :::::::	UUCUCCUUUUUCUUCUUCUUC	Cleavage	-25.3 kcal/mol
capa-miR529	CYVMV_AC1_Kahlilabad_RAV	5	543-563	GAAGAAGA-GAGAAGGAAGAA	:::: :.::.:: ::::::::	UUCUCCUUUUUCCUCUUCUUC	Cleavage	-26.1 kcal/mol
capa-miR529	CLCuMuV_AC1_Bastar_RAV	5	537-557	GAAGAA-GAGAGAAGGAAGAA	:::: :.::.:::: ::::::	UUCUCCUUUUUCUCGUUCUUC	Cleavage	-25.1 kcal/mol
capa-miR529	PaLCuV_AC1_Delhi_RAV	4.5	543-563	GAAGAAG-AGAGAAGGAAGAA	:::: :.:::::: :::::::	UUCUCCUUUCUCUUCUUCUUC	Cleavage	-27.7 kcal/mol
capa-miR838	ToLCNDV_AC1_Mahasamund_RAV	3	1074-1094	UCUUCUUCUUCUUCUUCUUCA	: ::::::::::::::. :	CGCAGAAGAAGAAGAAGAGCA	Cleavage	-30.2 kcal/mol
capa-miR838	ToLCNDV_AC1_Nautanwa_RAV	3	1032-1052	UCUUCUUCUUCUUCUUCUUCA	::::::.::::::::: :	CACAGAAGAGGAAGAAGAACA	Cleavage	-28.8 kcal/mol
capa-miR838	CLCuMuV_AC1_Bastar_RAV	5	352-372	UCUUCUUCUUCUUCUUCUUCA	::::: :. ::::.::.::	GGAAGAUCAGCAAGAGGAGGA	Translation	-26.6 kcal/mol
capa-miR838	ToLCNDV_AC2_Mahasamund_RAV	3	59-79	UCUUCUUCUUCUUCUUCUUCA	: ::::::::::::::. :	CGCAGAAGAAGAAGAAGAGCA	Cleavage	-30.2 kcal/mol
capa-miR838	ToLCNDV_AC2_Nautanwa_RAV	3	59-79	UCUUCUUCUUCUUCUUCUUCA	::::::.::::::::: :	CACAGAAGAGGAAGAAGAACA	Cleavage	-28.8 kcal/mol
capa-miR838	CLCuMuV_C4_Bastar_RAV	5	201-221	UCUUCUUCUUCUUCUUCUUCA	::::: :. ::::.::.::	GGAAGAUCAGCAAGAGGAGGA	Translation	-25.1 kcal/mol
capa-miR838	PaLCuB_betaC1_Bilaspur_RAV	5	17-37	UCUUCUUCUUCUUCUUCUUCA	:: ::::::..:: :.::	ACAACAAGAAGGGGAUGGAGU	Cleavage	-20.7 kcal/mol
capa-miR482	PaLCuV_AC1_Gorakhpur_av2	5	663-682	UCUUCCUUGUUCUUCCAUGU	:: : ::::::.: ::::	UGAUAGUAGAACAGGAAAGA	Cleavage	-26.3 kcal/mol
capa-miR482	PaLCuV_AC1_Bilaspur_RAV	5	582-601	UCUUCCUUGUUCUUCCAUGU	:: : ::::::.: ::::	UGAUAGUAGAACAGGAAAGA	Cleavage	-26.3 kcal/mol
capa-miR482	PaLCuV_AC1_Raipur_RAV	4.5	663-682	UCUUCCUUGUUCUUCCAUGU	:: : ::.::..::::::	UGAUAGUAGGACGGGGAAGA	Cleavage	-27.5 kcal/mol
capa-miR482	ToLCNDV_BV1_Durg_RAV	5	661-680	UCUUCCUUGUUCUUCCAUGU	::.: :::.::.: :::::	ACGUUCAAGGACGAAGAAGA	Cleavage	-21.0 kcal/mol
capa-miR1099	CLCuB_betaC1_Mahasamund_RAV	4.5	42-62	UUUAGCAAUGGUGAAUAUGUC	::: : ::::::: :::::	CACAAAGUCACCAUCGCUAAU	Cleavage	-23.0 kcal/mol
capa-miR1099	CLCuB_betaC1_Maharajganj_RAV	4.5	99-119	UUUAGCAAUGGUGAAUAUGUC	::: : ::::::: :::::	CACAAAGUCACCAUCGCUAAU	Cleavage	-23.0 kcal/mol
capa-miR5015b	PaLCuB_betaC1_Bilaspur_RAV	5	10-30	UCUUUUGUUGUUGUUGUUGUU	:: ::::::::: ::..:.	AAAUACAACAACAAGAAGGGG	Cleavage	-22.9 kcal/mol
capa-miR530b	ToLCNDV_AC1_Nautanwa_RAV	4	392-411	UGUUUUUGCAUCUGCAUCAU	: :::::: ::::::::..:.	ACGAUGCAUAUGCAAAGGCG	Cleavage	-27.7 kcal/mol

Table S3: identification of potential binding sites of papaya locus-derived capa-miRNAs predicted to bind the genes of begomovirus isolates using Tapirhybrid database.

miRNA_Acc.	Target_Acc.	MFE (kcal/mol)	Locus	MFE ratio (kcal/mol)
capa-miR1854-3p	ToLCNDV_AV2_Nautanwa_RAV	-19.7	112	0.47
capa-miR1854-3p	ToLCNDV_AV2_Mahasamund_RAV	-19.7	112	0.47
capa-miR838	PaLCuV_AV2_Gorakhpur_av1	-16.7	305	0.45
capa-miR838	PaLCuV_AV1_Gorakhpur_av1	-15.9	145	0.43
capa-miR1862g	ToLCBDB_betaC1_Delhi_RAV	-14.8	233	0.44
capa-miR1862g	ToLCBDB_betaC1_Bastar_RAV	-14.8	233	0.44
capa-miR1862f	ToLCBDB_betaC1_Delhi_RAV	-14.8	233	0.44
capa-miR1862f	ToLCBDB_betaC1_Bastar_RAV	-14.8	233	0.44
capa-miR529	PaLCuV_AV1_Raipur_RAV	-13.4	708	0.36
capa-miR529	PaLCuV_AV1_Raipur_RAV	-13.4	708	0.36
capa-miR902a-5p	PaLCuV_AC1_Gorakhpur_av1	-20.8	587	0.61
capa-miR902a-5p	CYVMV_AC1_Kahlilabad_RAV	-20.8	587	0.61
capa-miR902a-5p	PaLCuV_AC1_Durg_RAV	-20.8	587	0.61
capa-miR902a-5p	ToLCNDV_AC1_Nautanwa_RAV	-17.6	451	0.52
capa-miR530b	PaLCuV_AV1_Gorakhpur_av2	-16.8	497	0.49
capa-miR530b	ToLCNDV_AV1_Nautanwa_RAV	-15.8	189	0.46
capa-miR530b	ToLCNDV_AC3_Nautanwa_RAV	-14.8	337	0.43
capa-miR530b	ToLCNDV_AV1_Mahasamund_RAV	-14.5	667	0.42
capa-miR530b	ToLCNDV_AC3_Mahasamund_RAV	-13.1	279	0.38
capa-miR530b	ToLCNDV_BV1_Durg_RAV	-17.4	264	0.50
capa-miR530b	PaLCuA_Rep_Bilaspur_RAV	-17.1	631	0.50
capa-miR530b	PaLCVSA_Rep_Bastar_RAV	-17.7	625	0.51
capa-miR482	PaLCuV_AC1_Gorakhpur_av1	-24.4	355	0.64
capa-miR482	PaLCuV_C4_Gorakhpur_av1	-24.4	204	0.64
capa-miR482	CLCuV_AC1_Maharajganj_RAV	-25.9	349	0.68
capa-miR482	CLCuV_C4_Maharajganj_RAV	-25.9	204	0.68
capa-miR482	CYVMV_AC1_Kahlilabad_RAV	-24.4	355	0.64
capa-miR482	CYVMV_C4_Kahlilabad_RAV	-24.4	198	0.64
capa-miR482	PaLCuV_AC1_Durg_RAV	-24.4	355	0.64
capa-miR482	PaLCuV_C4_Durg_RAV	-24.4	198	0.64
capa-miR482	PaLCuV_AC1_Delhi_RAV	-20.3	355	0.53

capa-miR482	PaLCuV_C4_Delhi_RAV	-20.3	198	0.53
capa-miR482	CLCuMuV_AC1_Bastar_RAV	-27.4	349	0.72
capa-miR482	CLCuMuV_C4_Bastar_RAV	-27.4	198	0.72
capa-miR482	CLCuB_betaC1_Maharajganj_RAV	-10.7	150	0.28
capa-miR482	ToLCBDB_betaC1_Delhi_RAV	-21.9	46	0.57
capa-miR482	CLCuB_betaC1_Mahasamund_RAV	-11.9	87	0.31
capa-miR482	ToLCBDB_betaC1_Bastar_RAV	-21.9	46	0.57
capa-miR482	PaLCuA_Rep_Bilaspur_RAV	-21.1	623	0.55
capa-miR482	PaLCVSA_Rep_Bastar_RAV	-23.1	623	0.60
capa-miR166b	CLCuV_AC1_Maharajganj_RAV	-15.3	573	0.36
capa-miR166a	CLCuV_AC1_Maharajganj_RAV	-15.3	573	0.36
capa-miR5021	CLCuMuV_AV2_Bastar_RAV	-15.4	48	0.38
capa-miR5672	PaLCuV_AC1_Durg_RAV	-14.9	91	0.39
capa-miR5672	PaLCuV_AC1_Bilaspur_RAV	-14.9	10	0.39
capa-miR5672	CLCuMuV_AC1_Bastar_RAV	-16.8	707	0.44
capa-miR5658	PaLCuV_AV2_Gorakhpur_av2	-14.2	217	0.36
capa-miR5658	PaLCuV_AV1_Gorakhpur_av2	-14.2	57	0.36
capa-miR5658	CYVMV_AV2_Kahlilabad_RAV	-18.3	214	0.46
capa-miR5658	CYVMV_AV1_Kahlilabad_RAV	-18.3	54	0.46
capa-miR5658	PaLCuV_AV2_Durg_RAV	-18.3	214	0.46
capa-miR5658	PaLCuV_AV1_Durg_RAV	-18.3	54	0.46
capa-miR5658	PaLCuV_AV2_Raipur_RAV	-18.3	214	0.46
capa-miR5658	PaLCuV_AV1_Raipur_RAV	-18.3	54	0.46
capa-miR5658	ToLCNDV_AV2_Nautanwa_RAV	-15.4	217	0.39
capa-miR5658	ToLCNDV_AV1_Nautanwa_RAV	-15.4	57	0.39
capa-miR5658	PaLCuV_AV2_Bilaspur_RAV	-14.2	217	0.36
capa-miR5658	PaLCuV_AV1_Bilaspur_RAV	-14.2	57	0.36
capa-miR5658	ToLCNDV_AV2_Mahasamund_RAV	-11.8	217	0.30
capa-miR5658	ToLCNDV_AV1_Mahasamund_RAV	-11.8	57	0.30
capa-miR5658	ToLCNDV_AC2_Mahasamund_RAV	-18	5	0.45
capa-miR5658	ToLCNDV_AC1_Mahasamund_RAV	-18	1020	0.45
capa-miR5658	ToLCB_betaC1_Nautanwa_RAV	-10.3	143	0.26
capa-miR5658	PaLCuA_Rep_Bilaspur_RAV	-15.8	853	0.40

capa-miR776	CLCuV_AC1_Maharajganj_RAV	-20.2	909	0.58
capa-miR776	CLCuV_AC1_Maharajganj_RAV	-16.3	778	0.47
capa-miR776	PaLCuV_AC2_Durg_RAV	-10.2	218	0.29
capa-miR776	ToLCNDV_AC1_Nautanwa_RAV	-14.3	434	0.41
capa-miR776	ToLCNDV_AC2_Mahasamund_RAV	-10.2	218	0.29
capa-miR776	ToLCNDV_AC1_Mahasamund_RAV	-14.7	455	0.42
capa-miR776	PaLCVSA_Rep_Bastar_RAV	-15.6	905	0.45
capa-miR5565e	PaLCuV_AC2_Gorakhpur_av1	-15.2	92	0.44
capa-miR5565e	ToLCNDV_AV1_Nautanwa_RAV	-17.2	222	0.50
capa-miR3441.2	PaLCuV_AV1_Gorakhpur_av1	-16.5	661	0.47
capa-miR3441.2	PaLCuV_AC3_Gorakhpur_av1	-12.6	167	0.36
capa-miR3441.2	PaLCuV_AC2_Gorakhpur_av1	-12.6	312	0.36
capa-miR3441.2	CLCuV_AC3_Maharajganj_RAV	-12.6	167	0.36
capa-miR3441.2	CLCuV_AC2_Maharajganj_RAV	-12.6	318	0.36
capa-miR3441.2	CYVMV_AV1_Kahlilabad_RAV	-14.7	287	0.42
capa-miR3441.2	PaLCuV_AV1_Durg_RAV	-15.7	658	0.44
capa-miR3441.2	PaLCuV_AV1_Raipur_RAV	-15.7	661	0.44
capa-miR3441.2	PaLCuV_AV1_Delhi_RAV	-16.6	661	0.47
capa-miR3441.2	PaLCuV_AV1_Delhi_RAV	-14.7	287	0.42
capa-miR3441.2	PaLCuV_AC3_Delhi_RAV	-12.6	167	0.36
capa-miR3441.2	PaLCuV_AC2_Delhi_RAV	-12.6	312	0.36
capa-miR3441.2	PaLCuV_AC3_Bilaspur_RAV	-12.6	167	0.36
capa-miR3441.2	PaLCuV_AC2_Bilaspur_RAV	-12.6	312	0.36
capa-miR3441.2	ToLCNDV_AC3_Mahasamund_RAV	-12.6	173	0.36
capa-miR3441.2	ToLCNDV_AC2_Mahasamund_RAV	-12.6	312	0.36
capa-miR3441.2	CLCuMuV_AV1_Bastar_RAV	-13.2	658	0.37
capa-miR414	PaLCuV_AC1_Gorakhpur_av1	-9.9	294	0.27
capa-miR414	PaLCuV_C4_Gorakhpur_av1	-9.9	143	0.27
capa-miR414	ToLCNDV_AC1_Nautanwa_RAV	-16.4	350	0.46
capa-miR414	ToLCNDV_AC1_Mahasamund_RAV	-17	371	0.47
capa-miR414	CLCuMuV_AV1_Bastar_RAV	-14.1	235	0.39
capa-miR414	PaLCuV_AC1_Gorakhpur_av1	-9.9	294	0.27
capa-miR414	PaLCuV_C4_Gorakhpur_av1	-9.9	143	0.27

capa-miR414	ToLCNDV_AC1_Nautanwa_RAV	-16.4	350	0.46
capa-miR414	ToLCNDV_AC1_Mahasamund_RAV	-17	371	0.47
capa-miR414	CLCuMuV_AV1_Bastar_RAV	-14.1	235	0.39
capa-miR5140	ToLCBDB_betaC1_Delhi_RAV	-13.8	80	0.45
capa-miR5140	ToLCBDB_betaC1_Bastar_RAV	-13.8	80	0.45
capa-miR5015b	PaLCuV_AC1_Raipur_RAV	-14.3	626	0.43
capa-miR1536	ToLCNDV_BC1_Durg_RAV	-16.1	807	0.40

Table S4: identification of papaya locus-derived capa-miRNA-target pairs predicted to bind the genes of begomovirus isolates using RNA22 database.

<table>
<tr><th>miRNA_Acc.</th><th>Target_Acc.</th><th>Locus</th><th>MFE (kcal/mol).</th><th>Heteroduplex</th><th>P value</th></tr>
<tr><td>capa-miR5658</td><td>PaLCuV_AC1_Gorakhpur_av1</td><td>163</td><td>-15.30</td><td>GAACCTCATCTCCA-CGTCCT
||||| || |:||||
GTAGTAGTAGTAGTAGTAGGA</td><td>9.89E-2</td></tr>
<tr><td>capa-miR5658</td><td>PaLCuV_AC3_Delhi_RAV</td><td>126</td><td>-16.60</td><td>CAAAGACATCATCACCGTCCA
|||||||| |:|||
GTAGTAGTAGTAGTAGTAGGA</td><td>3.46E-2</td></tr>
<tr><td>capa-miR5658</td><td>PaLCuV_AC2_Delhi_RAV</td><td>271</td><td>-16.60</td><td>CAAAGACATCATCACCGTCCA
|||||||| |:|||
GTAGTAGTAGTAGTAGTAGGA</td><td>3.46E-2</td></tr>
<tr><td>capa-miR5658</td><td>ToLCB_betaC1_Nautanwa_RAV</td><td>88</td><td>-15.00</td><td>AATTATT-CCATCATCGTTCA
||:||: ||||||:|:|
GTAGTAGTAGTAGTAGTAGGA</td><td>1.43E-1</td></tr>
<tr><td>capa-miR530b</td><td>PaLCuV_AC1_Gorakhpur_av1</td><td>287</td><td>-15.00</td><td>GCTCGTCAGATGTCAAGGCC
||||||: ||::|
TACTACGTCTACGTTTTTGT</td><td>2.81E-1</td></tr>
<tr><td>capa-miR530b</td><td>PaLCuV_C4_Gorakhpur_av1</td><td>136</td><td>-15.00</td><td>GCTCGTCAGATGTCAAGGCC
||||||: ||::|
TACTACGTCTACGTTTTTGT</td><td>2.65E-1</td></tr>
<tr><td>capa-miR530b</td><td>PaLCuV_AC1_Gorakhpur_av2</td><td>392</td><td>-21.00</td><td>ATGATGCAGCAGCAGAGGCC
|||||||| |||:|::|
TACTACGTCTACGTTTTTGT</td><td>1.84E-2</td></tr>
<tr><td>capa-miR530b</td><td>PaLCuV_C4_Gorakhpur_av2</td><td>235</td><td>-21.00</td><td>ATGATGCAGCAGCAGAGGCC
|||||||| |||:|::|
TACTACGTCTACGTTTTTGT</td><td>1.72E-2</td></tr>
<tr><td>capa-miR530b</td><td>CYVMV_AC1_Kahlilabad_RAV</td><td>392</td><td>-17.00</td><td>ATGATGCTGCAGCAGAGGCC
||||||| |||:|::|
TACTACGTCTACGTTTTTGT</td><td>9.58E-2</td></tr>
<tr><td>capa-miR530b</td><td>CYVMV_C4_Kahlilabad_RAV</td><td>235</td><td>-17.00</td><td>ATGATGCTGCAGCAGAGGCC
||||||| |||:|::|
TACTACGTCTACGTTTTTGT</td><td>8.89E-2</td></tr>
<tr><td>capa-miR530b</td><td>PaLCuV_AC1_Durg_RAV</td><td>392</td><td>-21.00</td><td>ATGATGCAGCAGCAGAGGCC
|||||||| |||:|::|
TACTACGTCTACGTTTTTGT</td><td>1.84E-2</td></tr>
<tr><td>capa-miR530b</td><td>PaLCuV_C4_Durg_RAV</td><td>235</td><td>-21.00</td><td>ATGATGCAGCAGCAGAGGCC
|||||||| |||:|::|
TACTACGTCTACGTTTTTGT</td><td>1.72E-2</td></tr>
<tr><td>capa-miR530b</td><td>PaLCuV_AC1_Raipur_RAV</td><td>392</td><td>-17.00</td><td>ATGATGCTGCAGCAGAGGCC
||||||| |||:|::|
TACTACGTCTACGTTTTTGT</td><td>1.07E-1</td></tr>
<tr><td>capa-miR530b</td><td>PaLCuV_C4_Raipur_RAV</td><td>235</td><td>-17.00</td><td>ATGATGCTGCAGCAGAGGCC
||||||| |||:|::|
TACTACGTCTACGTTTTTGT</td><td>9.94E-2</td></tr>
<tr><td>capa-miR530b</td><td>PaLCuV_AC1_Bilaspur_RAV</td><td>311</td><td>-21.00</td><td>ATGATGCAGCAGCAGAGGCC
|||||||| |||:|::|
TACTACGTCTACGTTTTTGT</td><td>1.84E-2</td></tr>
<tr><td>capa-miR530b</td><td>PaLCuV_C4_Bilaspur_RAV</td><td>235</td><td>-21.00</td><td>ATGATGCAGCAGCAGAGGCC
|||||||| |||:|::|
TACTACGTCTACGTTTTTGT</td><td>1.72E-2</td></tr>
<tr><td>capa-miR529</td><td>PaLCuV_AC1_Gorakhpur_av2</td><td>543</td><td>-15.00</td><td>GTC-GCCTTTTTTATCTTCTTC
|| ||||:|: |||||||
AAGAAGGAAGAG--AGAAGAAG</td><td>1.64E-1</td></tr>
<tr><td>capa-miR529</td><td>CLCuV_AC1_Maharajganj_RAV</td><td>537</td><td>-19.20</td><td>TTCTCCCTTTTTATCTTCTTC
|||| ||||:|: |||||||
AAGAAGGAAGAG-AGAAGAAG</td><td>2.61E-1</td></tr>
<tr><td>capa-miR529</td><td>CYVMV_AC1_Kahlilabad_RAV</td><td>543</td><td>-19.60</td><td>TTCT-CCTTTTTCCTCTTCTTC
|||| ||||:| ||||||||
AAGAAGGAAGA--GAGAAGAAG</td><td>7.89E-3</td></tr>
<tr><td>capa-miR529</td><td>PaLCuV_AC1_Durg_RAV</td><td>543</td><td>-19.10</td><td>TTCTCCTTTTTCTTCTTCTTC
|||| |:||:||| |||||||
AAGAAGGAAGAGA-GAAGAAG</td><td>9.06E-3</td></tr>
</table>

<table>
<tr><td>capa-miR529</td><td>PaLCuV_AC1_Durg_RAV</td><td>548</td><td>-17.20</td><td>CTTTTTCTTCT-TCTTCTTT
|:||:||||| |||||||:
AAGAAGGAAGAGAGAAGAAG</td><td>9.06E-3</td></tr>
<tr><td>capa-miR529</td><td>PaLCuV_AC1_Raipur_RAV</td><td>543</td><td>-19.10</td><td>TTCTCCTTTTTCTTCTTCTTC
|||| |:||:||| ||||||
AAGAAGGAAGAGA-GAAGAAG</td><td>9.06E-3</td></tr>
<tr><td>capa-miR529</td><td>PaLCuV_AC1_Raipur_RAV</td><td>548</td><td>-17.20</td><td>CTTTTTCTTCT-TCTTCTTT
|:||:||||| |||||||:
AAGAAGGAAGAGAGAAGAAG</td><td>9.06E-3</td></tr>
<tr><td>capa-miR529</td><td>PaLCuV_AC1_Delhi_RAV</td><td>543</td><td>-21.70</td><td>TTCTCCTTTCTCTTCTTCTTC
|||| |:|||||| |||||||
AAGAAGGAAGAGA-GAAGAAG</td><td>4.9E-3</td></tr>
<tr><td>capa-miR529</td><td>PaLCuV_AC1_Bilaspur_RAV</td><td>462</td><td>-17.40</td><td>TTCT-CCTTTTTTATCTTCTTC
||| ||||:|: |||||||
AAGAAGGAAGAG--AGAAGAAG</td><td>2.01E-1</td></tr>
<tr><td>capa-miR5021</td><td>PaLCuV_AC1_Gorakhpur_av2</td><td>547</td><td>-17.90</td><td>CCTTTTTTATCTTCTTCTTT
|||:||: ||||||||||
AGAAGAAGAAGAAGAAGAAA</td><td>1.64E-1</td></tr>
<tr><td>capa-miR5021</td><td>CLCuV_AC1_Maharajganj_RAV</td><td>542</td><td>-15.20</td><td>CCTTTTT-ATCTTCTTCTTT
|||:|| ||||||||||
AGAAGAAGAAGAAGAAGAAA</td><td>2.61E-1</td></tr>
<tr><td>capa-miR5021</td><td>CYVMV_AC1_Kahlilabad_RAV</td><td>547</td><td>-19.60</td><td>CCTTTTTCCTCTTCTTCTTT
|||:||| ||||||||||
AGAAGAAGAAGAAGAAGAAA</td><td>7.89E-3</td></tr>
<tr><td>capa-miR5021</td><td>PaLCuV_AC1_Raipur_RAV</td><td>547</td><td>-23.70</td><td>CCTTTTTCTTCTTCTTCTTT
|||:||||||||||||||
AGAAGAAGAAGAAGAAGAAA</td><td>9.06E-3</td></tr>
<tr><td>capa-miR5021</td><td>PaLCuV_AC1_Durg_RAV</td><td>547</td><td>-23.70</td><td>CCTTTTTCTTCTTCTTCTTT
|||:||||||||||||||
AGAAGAAGAAGAAGAAGAAA</td><td>9.06E-3</td></tr>
<tr><td>capa-miR5021</td><td>PaLCuV_AC1_Delhi_RAV</td><td>547</td><td>-21.10</td><td>CCTTTCTCTTCTTCTTCTTT
||| ||||||||||||
AGAAGAAGAAGAAGAAGAAA</td><td>4.9E-3</td></tr>
<tr><td>capa-miR5021</td><td>PaLCuV_AC1_Bilaspur_RAV</td><td>466</td><td>-17.90</td><td>CCTTTTTTATCTTCTTCTTT
|||:||: ||||||||||
AGAAGAAGAAGAAGAAGAAA</td><td>2.01E-1</td></tr>
<tr><td>capa-miR5015b</td><td>PaLCuV_AC1_Gorakhpur_av2</td><td>391</td><td>-15.70</td><td>AATGATG-CAGCAGCAGAGGC
|| |:: ||:||:||:|:|
TTGTTGTTGTTGTTGTTTTCT</td><td>1.84E-2</td></tr>
<tr><td>capa-miR5015b</td><td>PaLCuV_AC1_Durg_RAV</td><td>391</td><td>-15.70</td><td>AATGATG-CAGCAGCAGAGGC
|| |:: ||:||:||:|:|
TTGTTGTTGTTGTTGTTTTCT</td><td>1.84E-2</td></tr>
<tr><td>capa-miR5015b</td><td>PaLCuV_C4_Delhi_RAV</td><td>227</td><td>-15.20</td><td>GACAGCTAATGATGCTGCAGAAGA
:|||:| || |:   :||:||||
TTGTTG-TTGTTG--TTGTTTTCT</td><td>1.2E-1</td></tr>
<tr><td>capa-miR5015b</td><td>PaLCuV_AC1_Bilaspur_RAV</td><td>310</td><td>-15.70</td><td>AATGATG-CAGCAGCAGAGGC
|| |:: ||:||:||:|:|
TTGTTGTTGTTGTTGTTTTCT</td><td>1.84E-2</td></tr>
<tr><td>capa-miR5015b</td><td>CLCuB_betaC1_Maharajganj_RAV</td><td>220</td><td>-16.30</td><td>AACAACAAGGCAAAGATAGAAGA
|||||| :||| :|:|:||||
TTGTTGT--TGTTGTTGTTTTCT</td><td>9.78E-2</td></tr>
<tr><td>capa-miR5015b</td><td>PaLCuB_betaC1_Bilaspur_RAV</td><td>10</td><td>-16.50</td><td>AAATACAACAACAAGAAGGGG
||||||||| ||::|:
TTGTTGTTGTTGTTGTTTTCT</td><td>5.81E-2</td></tr>
<tr><td>capa-miR838</td><td>CLCuMuV_AC1_Bastar_RAV</td><td>349</td><td>-17.30</td><td>GATGGAAGATCAGCAAGAGGAGGA
:||||| || ||||:||:||
ACTTCTTCT--TC-TTCTTCTTCT</td><td>1.44E-1</td></tr>
<tr><td>capa-miR838</td><td>CLCuMuV_C4_Bastar_RAV</td><td>198</td><td>-17.30</td><td>GATGGAAGATCAGCAAGAGGAGGA
:||||| || ||||:||:||
ACTTCTTCT--TC-TTCTTCTTCT</td><td>1.34E-1</td></tr>
<tr><td>capa-miR838</td><td>ToLCNDV_BV1_Durg_RAV</td><td>662</td><td>-17.60</td><td>CGTTCAAGGACGAAGAAGAAGA
|||:| ||||||||||
ACTTCTTCTT-CTTCTTCTTCT</td><td>4.95E-2</td></tr>
<tr><td>capa-miR482</td><td>PaLCuV_AC1_Raipur_RAV</td><td>663</td><td>-17.10</td><td>TGATAGTAGGACGGGGAAGA
||:||::||||||
TGTACCTTCTTGTTCCTTCT</td><td>3.71E-1</td></tr>
<tr><td>capa-miR482</td><td>CLCuV_AC1_Maharajganj_RAV</td><td>654</td><td>-20.40</td><td>GGGTGAGAGTAGAACAGGGAAGA
: :|| || ||||||:||||||
TGTAC-CT--TCTTGTTCCTTCT</td><td>1.64E-1</td></tr>
<tr><td>capa-miR902a-5p</td><td>PaLCuV_AC3_Delhi_RAV</td><td>247</td><td>-18.80</td><td>TTAAGGGTCTTCAAGACC
|||::|||:||||||</td><td>1.22E-1</td></tr>
</table>

<table>
<tr><td></td><td></td><td></td><td></td><td>ACTTCTTAGAGGTTCTGT</td><td></td></tr>
<tr><td>capa-miR902a-5p</td><td>PaLCuV_AC2_Delhi_RAV</td><td>392</td><td>-18.80</td><td>TTAAGGGTCTTCAAGACC
|||::|||:||||||
ACTTCTTAGAGGTTCTGT</td><td>1.28E-1</td></tr>
<tr><td>capa-miR1854-3p</td><td>PaLCuV_AC2_Delhi_RAV</td><td>76</td><td>-15.60</td><td>GGGACCAGACGTCGCAGAGTTGA
||||:| :| ||:|:|:||
AAGTGGTTT-AGGGGTTTTAGCT</td><td>5.33E-2</td></tr>
<tr><td>capa-miR1536</td><td>PaLCuV_AC1_Gorakhpur_av1</td><td>534</td><td>-16.50</td><td>GGTTTATGTATGTTTCTCCTT
:|::| |||:||||||:
GTTTGTGTAGACAGAGAGGAG</td><td>1.95E-1</td></tr>
<tr><td>capa-miR5140</td><td>CLCuMuV_AC1_Bastar_RAV</td><td>132</td><td>-15.10</td><td>CTGCAGAGAGCTTCACCAAA
||:| |||||||||
TTGGTTT--AGAAGTGGTTT</td><td>1.96E-2</td></tr>
<tr><td>capa-miR5565e</td><td>ToLCNDV_BC1_Durg_RAV</td><td>729</td><td>-16.40</td><td>TCCGTCAGCATCACAAGCAG
|| ||||:|| |||:||:
AGTCAGTTGT-TGGTTTGTT</td><td>3.64E-1</td></tr>
</table>
