# Supplementary figures and images for: In silico identification of papaya genome-encoded microRNAs to target begomovirus genes in papaya leaf curl disease
Source: Front Microbiol. 2024 Mar 21;15:1340275. doi: 10.3389/fmicb.2024.1340275 (PMC11008722; doi:10.3389/fmicb.2024.1340275)

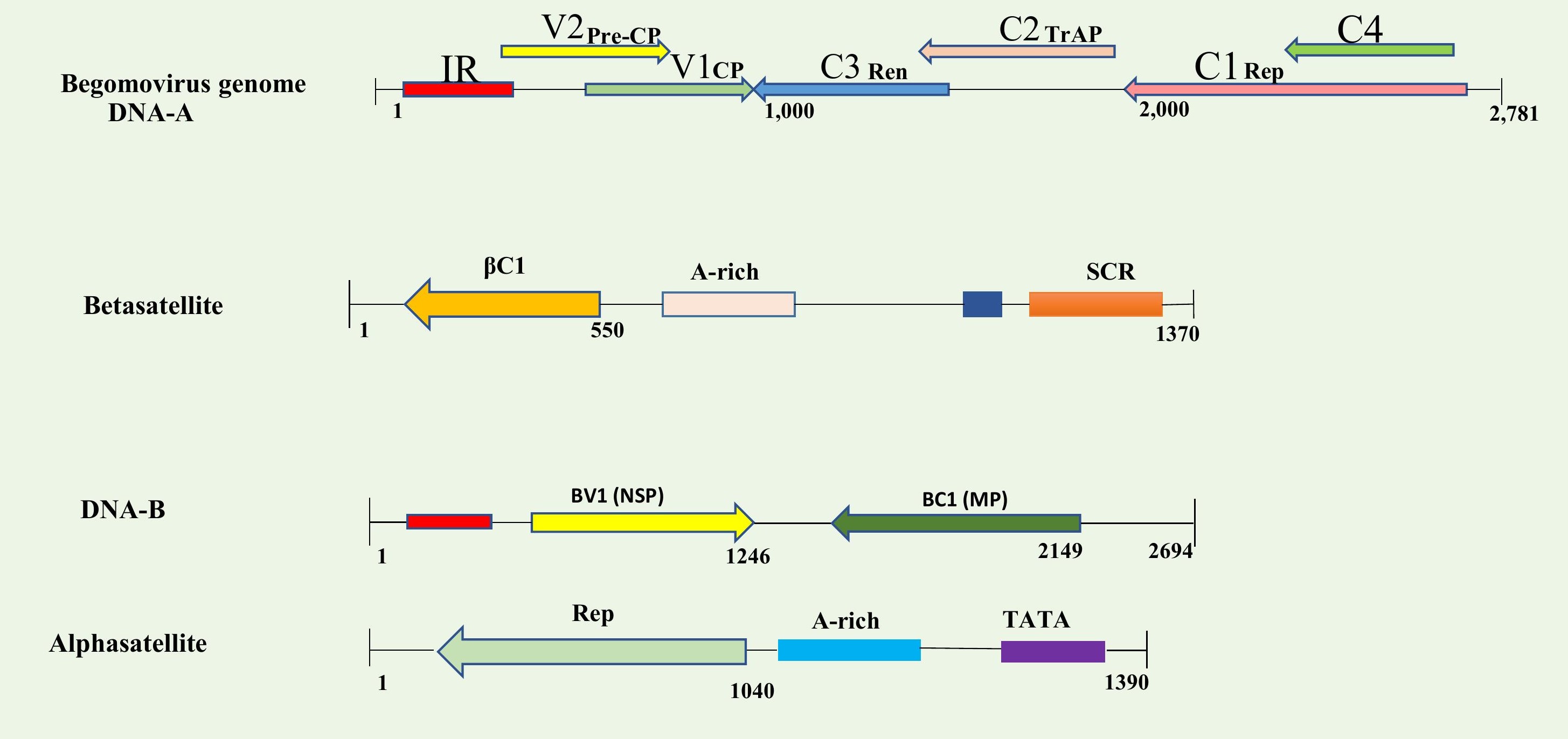

Supplement: Supplementary file 2 [file Image_1.jpeg]
